# AmiP from hyperthermophilic *Thermus parvatiensis* prophage is a thermoactive and ultrathermostable peptidoglycan lytic amidase

**DOI:** 10.1002/pro.4585

**Published:** 2023-02-15

**Authors:** Andrius Jasilionis, Magdalena Plotka, Lei Wang, Sebastian Dorawa, Joanna Lange, Hildegard Watzlawick, Tom van den Bergh, Bas Vroling, Josef Altenbuchner, Anna‐Karina Kaczorowska, Ehmke Pohl, Tadeusz Kaczorowski, Eva Nordberg Karlsson, Stefanie Freitag‐Pohl

**Affiliations:** ^1^ Division of Biotechnology, Department of Chemistry Lund University Lund Sweden; ^2^ Laboratory of Extremophiles Biology, Department of Microbiology, Faculty of Biology University of Gdansk Gdansk Poland; ^3^ Institute of Biomedical Genetics University of Stuttgart Stuttgart Germany; ^4^ Bio‐Prodict BV Nijmegen The Netherlands; ^5^ Collection of Plasmids and Microorganisms, Faculty of Biology University of Gdansk Gdansk Poland; ^6^ Department of Biosciences Durham University Durham UK; ^7^ Department of Chemistry Durham University Durham UK

**Keywords:** adaptation, Amidase_3 catalytic domain, peptidoglycan lytic amidases, thermoactivity, thermostability, *Thermus* prophage

## Abstract

Bacteriophages encode a wide variety of cell wall disrupting enzymes that aid the viral escape in the final stages of infection. These lytic enzymes have accumulated notable interest due to their potential as novel antibacterials for infection treatment caused by multiple‐drug resistant bacteria. Here, the detailed functional and structural characterization of *Thermus parvatiensis* prophage peptidoglycan lytic amidase AmiP, a globular Amidase_3 type lytic enzyme adapted to high temperatures is presented. The sequence and structure comparison with homologous lytic amidases reveals the key adaptation traits that ensure the activity and stability of AmiP at high temperatures. The crystal structure determined at a resolution of 1.8 Å displays a compact α/β‐fold with multiple secondary structure elements omitted or shortened compared with protein structures of similar proteins. The functional characterization of AmiP demonstrates high efficiency of catalytic activity and broad substrate specificity toward thermophilic and mesophilic bacteria strains containing Orn‐type or DAP‐type peptidoglycan. The here presented AmiP constitutes the most thermoactive and ultrathermostable Amidase_3 type lytic enzyme biochemically characterized with a temperature optimum at 85°C. The extraordinary high melting temperature *T*_m_ 102.6°C confirms fold stability up to approximately 100°C. Furthermore, AmiP is shown to be more active over the alkaline pH range with pH optimum at pH 8.5 and tolerates NaCl up to 300 mM with the activity optimum at 25 mM NaCl. This set of beneficial characteristics suggests that AmiP can be further exploited in biotechnology.

## Abstract 2

PDB Code(s): 7B3N.

## 1 INTRODUCTION

The release of viral progeny in the terminal phase of phage infection is mediated by a wide range of bacteriophage lytic enzymes. These enzymes are capable of hydrolyzing bacterial peptidoglycan components (Cahill & Young, 2019; Freitag‐Pohl et al., 2019; Plotka et al., 2015, 2019). Cell wall disrupting enzymes also designated endolysins have been extensively studied as an alternative or to complement current antimicrobial strategies and for biotechnology applications (Briers, 2019; Brives & Pourraz, 2020; Guo et al., 2020; Oliveira et al., 2012; Trudil, 2015). Robust enzymes from extremophiles with high stability at increased temperatures, at extreme pH or high salt concentrations are of particular interest for applications in the bioeconomy. Endolysins can be divided into three groups (i) peptidases that cleave the amide bonds between amino acids; (ii) glycosidases capable of hydrolysing glycosidic linkage; and (iii) amidases cleaving the amide bond between N‐acetylmuramic acid and L‐alanine at the N‐terminal portion of the stem peptide in peptidoglycan mesh (Vermassen et al., 2019).

Cell wall amidases are zinc‐dependent lytic hydrolases and are divided based on their catalytic domain type into the families Amidase_2 (Pfam PF01510), Amidase_3 (Pfam PF01520) and Amidase_5 (Pfam 05382) (Büttner et al., 2015). The Amidase_2 and Amidase_3 catalytic domains are structurally well‐characterized. Their functions are cell separation and cell wall lysis, respectively (Do et al., 2020). The functional importance of lytic amidases encoded in bacterial as well as viral genomes is associated with effective cell lysis by peptidoglycan disruption under certain conditions during infection or virion release. These enzymes contain cell wall binding domains (CBDs; Oliveira et al., 2013). Lytic amidases that lack CBDs but consist of a sole catalytic domain are globular lytic amidases. Cell wall amidases from bacteriophages feature a wide variety of domain organizations, including a diverse group of globular Amidase_3 type lytic amidases (Vermassen et al., 2019). The Amidase_3 domain fold is characterized as a central six‐stranded and mostly parallel β‐sheet surrounded by helices. The rear side interacts with three stretched helices α1, α3, and α5 forming the hydrophobic core. The β‐sheet front is solvent accessible and forms a wide cleft defined by loops (Büttner et al., 2015; Kumar et al., 2013). The Zn^2+^ ion in the catalytic center of the Amidase_3 domain is coordinated by two conserved histidines and a glutamate residue (Firczuk & Bochtler, 2007).

Peptidoglycan lytic amidases from mesophilic organisms containing the Amidase_3 catalytic domain have been well‐characterized with the aim to exploit the lytic activity toward clinically relevant bacteria as well as comprehend the molecular basis of substrate specificity. The Cwp6 cell wall protein from *Clostridioides difficile* for example has been shown to consists of five domains including the C‐terminal Amidase_3 domain (Bradshaw et al., 2018; Usenik et al., 2017). Structurally related Amidase_3 type lytic enzymes from a range of phages infecting *Clostridioides* species (ϕCD27, ϕATCC 8074‐B1, ϕCTP1, ϕCP26F, ϕCP39O) were investigated as potential antimicrobial agents albeit with a narrow substrate specificity (Mayer et al., 2010, 2011; Seal, 2013; Simmons et al., 2010). The endolysin CD27L specificity toward gram‐positive bacteria in particular *C. difficile* strains was optimized by CBD truncation resulting in a variant with increased lytic activity and effectiveness (Dunne et al., 2014; Mayer et al., 2011). To broaden the substrate specificity, the CBD of endolysin CD27L was fused with the Amidase_3 catalytic domain of endolysin CS74L encoded by lytic bacteriophage ATCC 8074‐B1 infecting *Clostridioides* strains. However, the substrate specificity of this chimeric protein was similar to that of CS74L (Mayer et al., 2012).

The Amidase_3 type lytic enzymes from cyanobacteria are capable of forming nanopores that perforate the peptidoglycan (Lehner et al., 2013). The functional importance of AmiC2 cell wall amidase from *Nostoc punctiforme* for nanopore formation in filamentous cyanobacteria is similar to the comprehensively characterized cell separation amidases AmiB from *Bartonella henselae* (Yang et al., 2012) and AmiC from *Escherichia coli* (Büttner et al., 2016; Lehner et al., 2011; Rocaboy et al., 2013). Interestingly, some globular lytic amidases possess intramolecular activity regulatory elements as was shown for the Rv3717 peptidoglycan amidase from *Mycobacterium tuberculosis*, which is autocontrolled by a disulfide bridge located in a β‐hairpin adjacent to the active site (Prigozhin et al., 2013). Rv3717, a broad substrate specific amidase, consists of a sole catalytic domain hydrolysing peptidoglycan fragments (muramyl dipeptides; Kumar et al., 2013). Amidase_3 enzyme activity can also be controlled by oligomeric states as has been shown for the CD27L, CS74L, and CTP1L, where the change of oligomer state triggers the cleavage of the C‐terminal domain, leading to the release of the catalytic domain what in consequence causes the effective bacterial cell lysis (Dunne et al., 2014; Dunne et al., 2016).

Several of the modular autolysins from *Bacillus* species as well as endolysins from corresponding bacteriophages contain an Amidase_3 catalytic domain. *Bacillus anthracis* autolysin AmiBA2446 acts predominantly as a dimer and lyses either *B. anthracis* cells, germinating spores or *Bacillus cereus* and *Bacillus thuringiensis* (Mehta et al., 2013). The moderately thermostable endolysin PlyTB40 from phage TsarBomba and endolysin LysPBC4 from ϕPBC4 have demonstrated lytic activity toward *B. cereus* strains (Etobayeva et al., 2018; Na et al., 2016).

Peptidoglycan lytic amidases containing Amidase_3 domains from thermophilic microorganisms remain sporadically investigated (Aevarsson et al., 2021; Fernández‐Ruiz et al., 2018). The phage E2 infecting thermophile deep‐sea *Geobacillus* sp. E263 encodes the thermostable Amidase_3 type lysin GVE2, which is involved in holin interactions as well as cell lysis (Jin et al., 2013; Ye & Zhang, 2008). This enzyme and endolysin PlyGspY412 encoded in *Geobacillus* sp. Y412MC61 prophage were used for the construction of putatively thermoactive and thermostable endolysins with desired substrate specificity. Although these chimeric amidases demonstrated higher thermostability, they were not thermoactive (Swift et al., 2015, 2019).

Since globular lytic amidases from bacteriophages infecting thermophilic microorganisms, in particular Amidase_3 type lytic enzymes remain underinvestigated, the characteristic features ensuring thermostability and thermoactivity need to be identified. These robust lytic enzymes could be implemented for clinical needs as well as exploited in biotechnology—in either the native form or after alteration by protein engineering (Oliveira et al., 2018; São‐José, 2018). The obvious potential of the Amidase_3 catalytic domain to be used for chimeric modular enzyme construction also outlines the necessity to target these enzymes to investigate the structural basis of thermostability and substrate promiscuity (Gerstmans et al., 2018). Therefore, the putative cell wall hydrolase AmiP from hyperthermophilic gram‐negative bacteria *Thermus parvatiensis* DSM 21745 prophage was selected for detailed characterization. The AmiP characterization included extensive biophysical, biochemical, and structural characterization accompanied by a comprehensive lytic activity examination.

## 2 RESULTS

### 2.1 AmiP gene identification and sequence analysis

*Thermus parvatiensis* (RL) DSM 21745 genome annotation reveals two prophage sequences (GenBank CP014141.1; Dwivedi et al., 2012, 2015; Tripathi et al., 2017). The *amiP* gene (GenBank AMA76298.1) sequence was annotated (locus AV541_09585) in a 23.28 kb prophage sequence (1751557–1774840 region) as encoding a putative cell wall hydrolase. The prophage sequence was characterized as an intact integron restricted by *attL* and *attR* integrase attachment sites.

The coding region of the *amiP* gene is 534 bp with an overall GC‐content of 67.8%. The gene encodes for the AmiP protein of 177 amino acids with an estimated isoelectric point at pH 9.75 and a calculated molecular mass of 19,171 Da. Signal peptide or transmembrane sequence regions are not predicted in the AmiP sequence. Homology searches show that the AmiP sequence is identical to sequences of the putative peptidoglycan amidases annotated in draft genomes of *Thermus scotoductus* strains (Wilpiszeski et al., 2019) and differs only by one amino acid from the putative amidase sequence (RefSeq WP_124105045.1) annotated in *T. thermophilus* TTHNAR1 genome. Furthermore, the putative peptidoglycan amidase (GenBank ACV05037.1) annotated in the genome of *Thermus* tectivirus P23‐77 infecting *T. thermophilus* DSM 674 shares 82.3% sequence identity with the AmiP sequence. Significant sequence identities between 43% and 79% are observed aligning AmiP with putative peptidoglycan amidases from genomes of various *Meiothermus* and *Deinococcus* strains. The sequence analysis shows that the N‐acetylmuramoyl‐l‐alanine amidase domain (Amidase_3, Pfam PF01520), which includes Ile^11^–Gly^147^, is the only domain predicted in the AmiP sequence. The predicted binding site for the catalytic Zn^2+^ ion in AmiP based on multiple sequence alignment comprises His^17^, His^85^, and Glu^32^ with Glu^145^ acting as the catalytic residue (Figure S1). This suggests a catalytic site architecture and reaction mechanism characteristic for the Amidase_3 domain (Lu et al., 2020).

The analysis of the amino acid composition shows an underrepresentation of thermolabile residues in the AmiP sequence compared to related lytic amidases encoded by mesophilic bacteria (Table S1). Thermolabile amino acids such as Asn, Gln, Met, and Cys, respectively (Pohl et al., 2002), appear less frequently, while Arg and Pro residues, which tend to be overrepresented in thermostable proteins are more frequent in the AmiP sequence. The overall amino acid composition of the catalytic domain of the AmiP sequence corresponds to residue frequencies characteristic for proteins from hyperthermophilic microorganisms (Hait et al., 2020; Taylor & Vaisman, 2010; Walden et al., 2004).

### 2.2 AmiP sequence phylogenetic analysis

**FIGURE 1:**
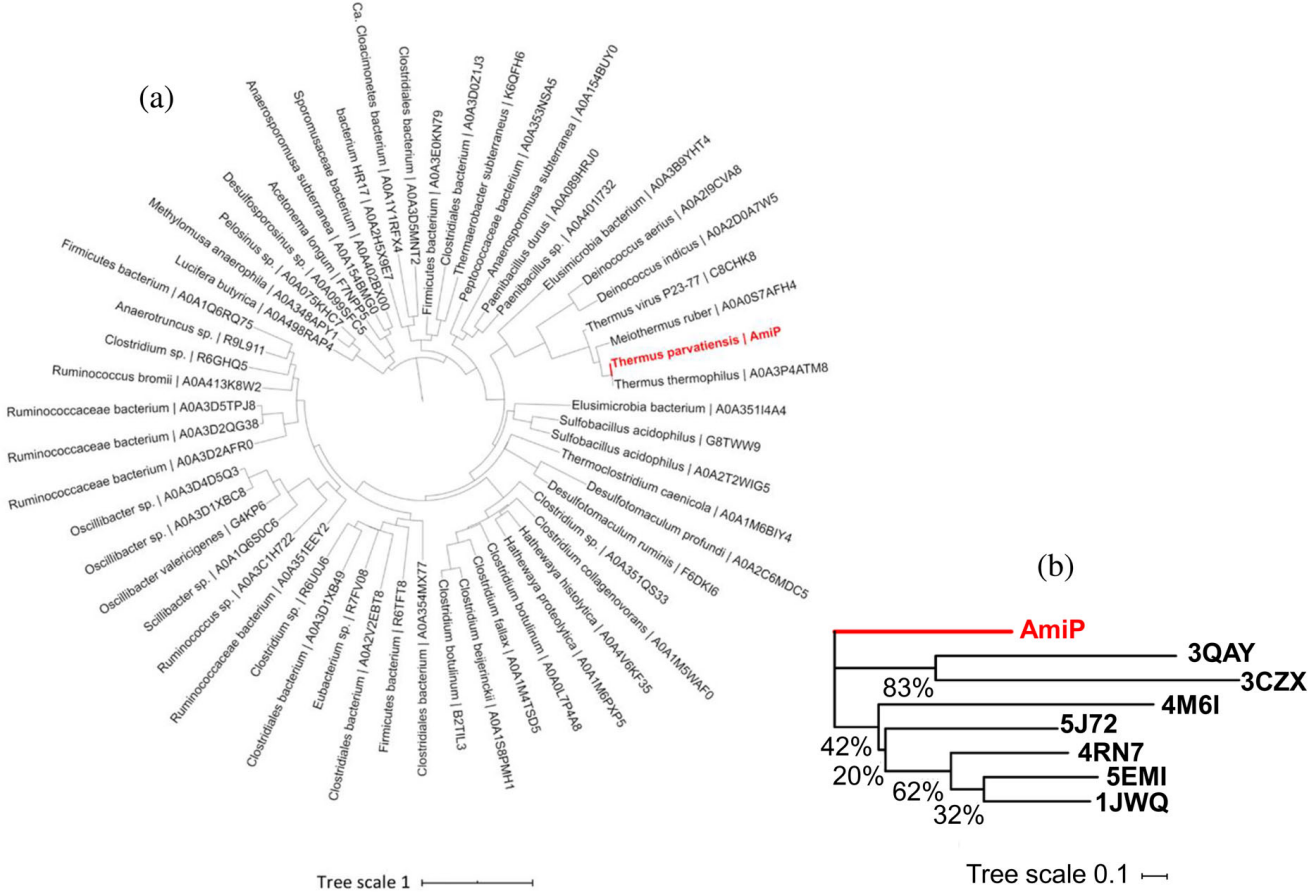
Phylogenetic analysis of AmiP. (a) Phylogenetic position of AmiP in a clade of evolutionarily most related peptidoglycan amidases. Protein sequences are indicated as accessible via UniProtKB. (b) Phylogenetic relationships of the AmiP sequence with sequences of structurally characterized homologues. PDB 3QAY (Mayer et al., 2011)—catalytic domain of endolysin CD27L from *Clostridioides difficile* phage ϕCD27 (UniProtKB B6SBV8), PDB 3CZX (Zhang et al., n.d.)—putative N‐acetylmuramoyl‐l‐alanine amidase from *Neisseria meningitidis* (UniProtKB Q9JZE9), PDB 4M6I (Prigozhin et al., 2013)—Rv3717 peptidoglycan amidase from *Mycobacterium tuberculosis* (UniProtKB O69684), PDB 5J72 (Usenik et al., 2017)—amidase domain of Cwp6 cell wall protein from *Clostridioides difficile* (UniProtKB Q183L9), PDB 4RN7 (Tan et al., n.d.)—catalytic domain of putative N‐acetylmuramoyl‐l‐alanine amidase from *Clostridioides difficile*(UniProtKB Q183J9), PDB 5EMI (Büttner et al., 2016)—catalytic domain of AmiC2 cell wall amidase from *Nostoc punctiforme* (UniProtKB B2J2S4), PDB 1JWQ (Yamane et al., 2003)—catalytic domain of CwlV cell wall amidase from *Paenibacillus polymyxa* subsp. *colistinus* (UniProtKB Q9LCR3). The bootstrap consensus values are indicated at nodes, respectively.

The evolutionary relationship of AmiP within the Amidase_3 type lytic amidases family based on multiple sequence alignment with all homologous protein sequences accessible with UniProtKB and sequence grouping localizes AmiP in a clade together with peptidoglycan amidases encoded by *Deinococcus* and *Thermus* phylum bacteria strains (Figure 1a). The putative amidase from *Thermus* tectivirus P23‐77 is also localized among the closest AmiP homologues with bootstrap consensus value above 88% for the corresponding node. Divergence into clades with amidases encoded by only *Deinococcus* strains and a clade with amidases from *Thermus* or *Meiothermus* strains is confirmed by bootstrap consensus values above 99% and 87% for the corresponding nodes, respectively. Amidases evolutionarily related with AmiP in genomes of *Deinococcus* strains are not encoded in prophage sequences. In contrast, respective genes in genomes of *Thermus* strains or *Meiothermus ruber* H328 are encoded in prophage sequences (data not presented). The phylogenetic relationships of AmiP with structurally characterized homologues are comparatively distant, yet a traceable evolutionary relationship between peptidoglycan amidases was determined (Figure 1b).

### 2.3 Protein production and purification

The native *amiP* gene sequence was expressed in *Escherichia coli* using a rhamnose‐inducible expression system (Wegerer et al., 2008) at excellent yield obtaining almost 12 mg of recombinant protein per liter of culture. Protein production had no toxic effect on expression culture growth. AmiP remained soluble in expression strain cells. Nickel affinity chromatography was successfully applied for AmiP purification to near homogeneity. The identity, integrity and purity of the protein were confirmed by ESI‐MS (Figure S2) and SDS‐PAGE (Figure S3). AmiP is stable and non‐aggregation‐prone in lysis and elution as well as reaction buffers.

### 2.4 AmiP biochemical activity characterization

**FIGURE 2:**
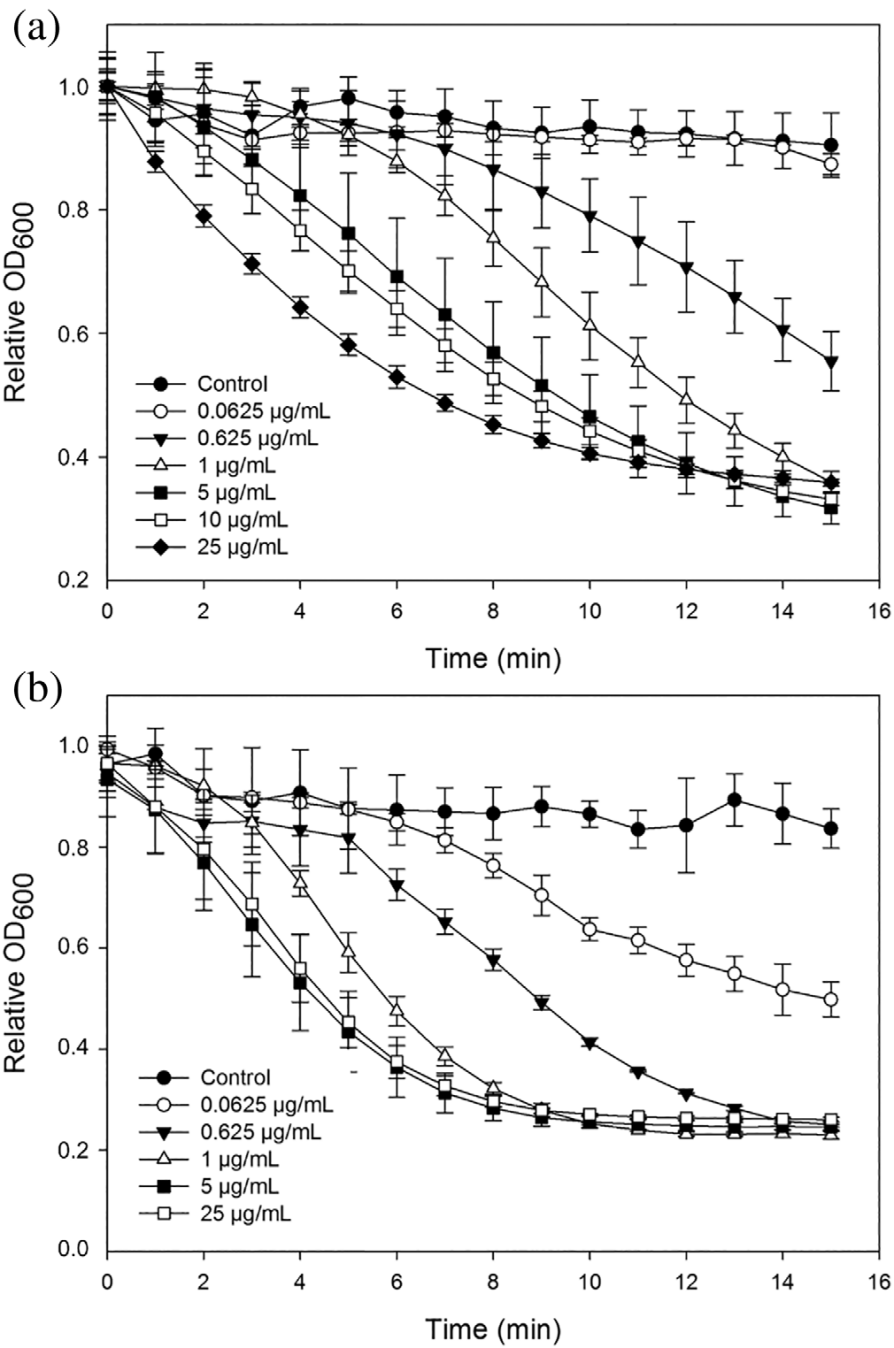
Dose range of AmiP catalytic activity measurement performing a turbidity reduction assay in a reaction mixture containing enzyme in the concentration range of 0.0625–25.0 μg/mL at 60°C. (a) Lytic activity determination with chloroform treated *Thermus thermophilus* DSM 579^T^ substrate. (b) Lytic activity determination with chloroform treated *Thermus parvatiensis* DSM 21745. The cell substrate suspension in reaction buffer served as negative control (black circles). Values represent the mean ± standard deviation (*n* = 3)

The catalytic activity of AmiP was confirmed performing a turbidity reduction assay at 60°C with chloroform treated *T. thermophilus* DSM 579^T^ substrate used for dose range and biochemical activity characterization as well as *T. parvatiensis* DSM 21745 substrate also used for dose range characterization. AmiP activity, as expected, increases proportionally to protein concentration in the range of 0.0625–25.0 μg/mL with the saturation of lysis with *T. thermophilus* DSM 579^T^ and *T. parvatiensis* DSM 21745 substrates reached at 25 μg/mL after 11 min of incubation and 1 μg/mL after 9 min of incubation, respectively (Figure 2).

**FIGURE 3:**
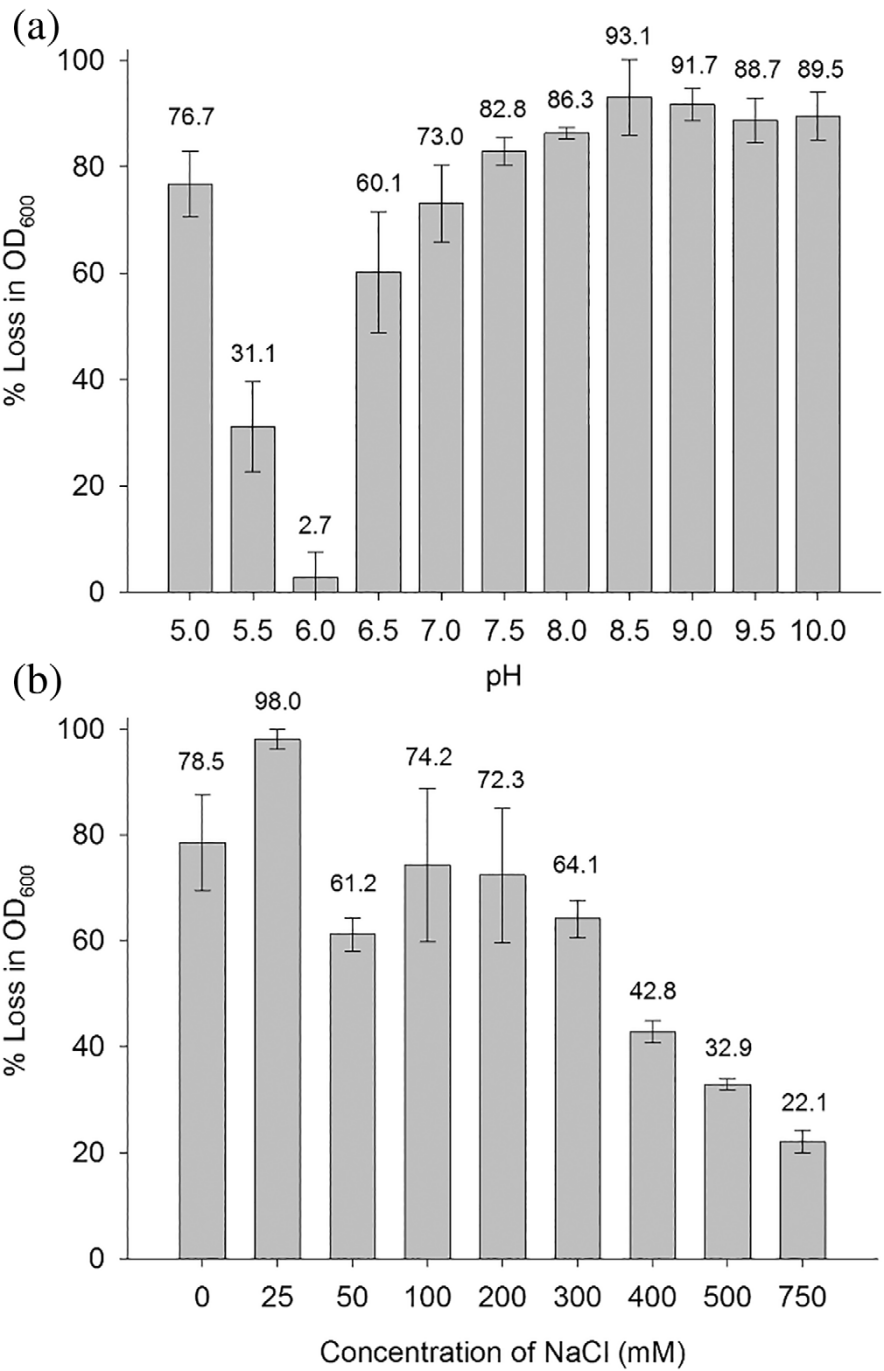
The effect of pH and NaCl concentration on AmiP lytic activity at 60°C. (a) The pH optimum of AmiP activity determination. (b) The optimal concentration of NaCl for AmiP activity determination. Relative activities are expressed as the percentage of the highest lytic activity determined. Values represent the mean ± standard deviation (*n* = 3) and are normalized to the highest activity measured in a single experiment

The lytic activity of AmiP was determined over a wide pH range of pH 5.0–10.0. Over the alkaline pH 7.5–10.0 enzyme activity exceeded 80% with pH optimum at pH 8.5 (Figure 3a). The activity of AmiP decreased gradually by roughly 10% from pH 7.5 to pH 7.0 and again from pH 7.0 to pH 6.5. The enzyme was barely active at pH 6.0, however AmiP's lytic activity increased sharply at pH 5.5 reaching approximately 77% at pH 5.0. The effect of NaCl concentration on AmiP activity was determined with NaCl concentrations in a range of 0–750 mM. The activity optimum was at 25 mM NaCl (Figure 3b). The optimal concentration of NaCl was increasing the enzyme activity by approximately 20%, while the addition of 50–300 mM NaCl in the assay showed a minor inhibiting effect on the lytic activity. Further addition of NaCl (400–750 mM) decreased the lytic activity of AmiP more significantly.

**FIGURE 4:**
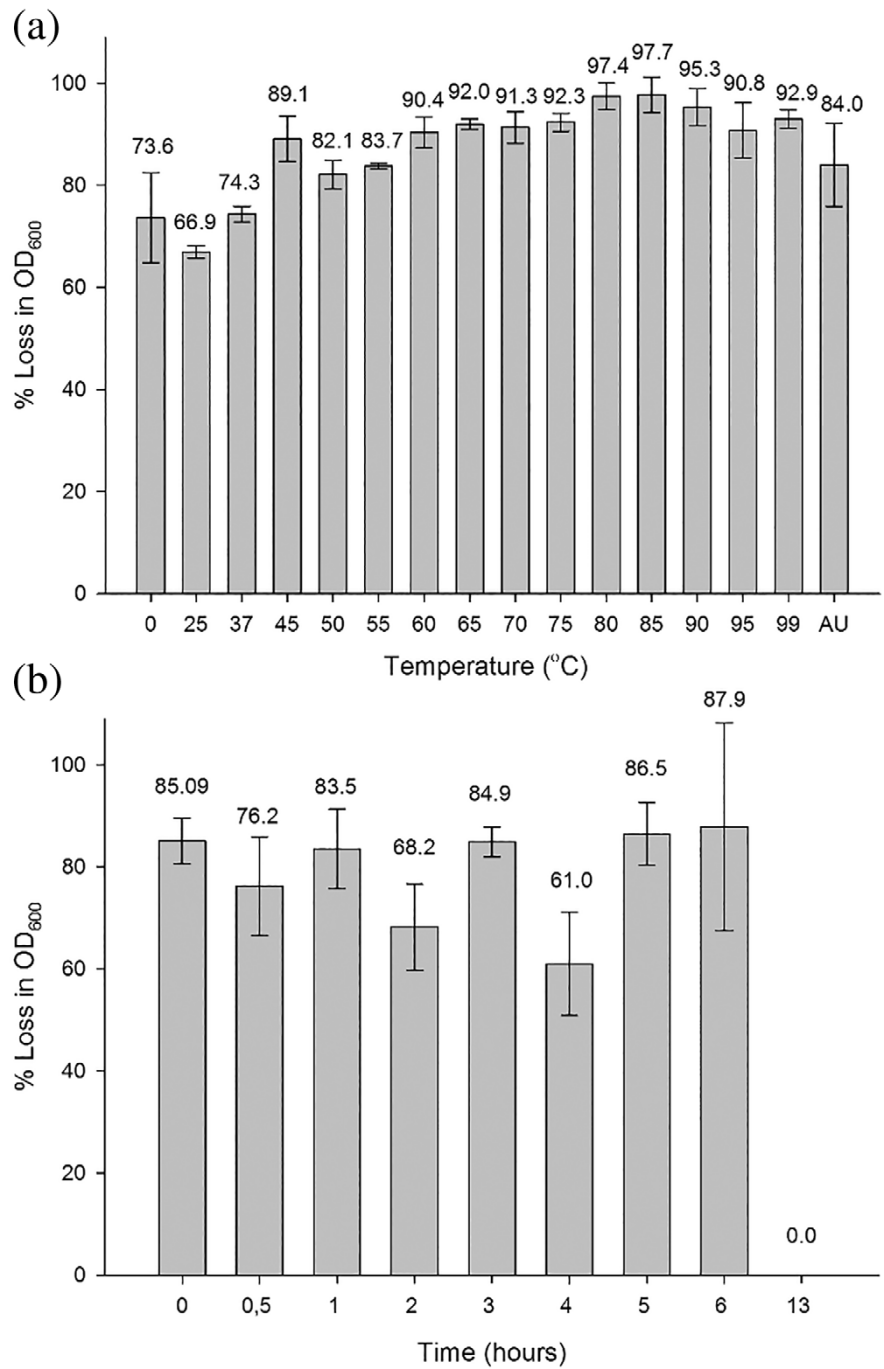
Thermoactivity and thermostability of AmiP amidase. (a) Thermoactivity of AmiP by measuring residual lytic activity at 60°C after enzyme preincubation for 30 min at indicated temperature or autoclavation for 20 min at 121°C. AU, autoclavation. (b) Thermostability of AmiP by measuring residual lytic activity at 60°C after enzyme preincubation for indicated time at 99°C. Relative residual activities are expressed as the percentage of the highest residual lytic activity determined. Values represent the mean ± standard deviation (*n* = 3) and are normalized to the highest activity measured in a single experiment

The thermoactivity of AmiP was confirmed by measuring the residual lytic activity at 60°C after preincubation over a wide temperature range of 0–99°C as well as after autoclavation. The residual activity was reaching 67%–89% after AmiP was preincubated for 30 min at 0–55°C (Figure 4a). While the enzyme almost retained full activity after preincubation for 30 min at 60–99°C as the measured residual activity was 90–98%. The temperature optimum was determined at 85°C. AmiP withstood even autoclavation for 20 min at 121°C with residual activity of approximately 84%. The extraordinary high thermostability of AmiP was also confirmed by prolonged incubation of the enzyme at 99°C (Figure 4b). AmiP was thermostable for 0.5–6 h at 99°C, though no residual lytic activity was detected after preincubation for 13 h at 99°C.

### 2.5 AmiP thermostability and thermoaggregation characterization

The fold stability was assayed over a temperature range of 20–110°C by nanoscale differential scanning fluorimetry and backreflection technology, respectively. The melting temperature *T*_m_ of AmiP at high enzyme concentration in optimal reaction buffer supplemented with 5% (vol/vol) glycerol was 102.6 ± 0.2°C (Figure S4A,B). AmiP demonstrated fold stability up to approximately 100°C, while a well‐defined thermal unfolding transition was observed between 100 and 105°C leading to irreversible denaturation. The simultaneously determined AmiP mid‐aggregation temperature *T*_agg_ 102.0 ± 0.1°C (Figure S4C) corresponds to the AmiP melting temperature. The AmiP aggregation onset temperature was approximately 100°C, observing irreversible aggregation up to 110°C. Thermostability and thermoaggregation characterization confirms AmiP's adaptation to high temperatures.

### 2.6 AmiP substrate specificity

The lytic activity of AmiP was further explored performing a turbidity reduction assay at 37 or 60°C with selected mesophilic and thermophilic bacteria strain substrates (Table 1). The highest lytic activity, as expected, was observed with other gram‐negative species, as *Thermus* strains. The *Thermus flavus* strain was lysed more effectively by approximately 5% than AmiP's native host *T. parvatiensis*. *T. thermophilus* and *T. scotoductus* strains were lysed less effective by approximately 10% and 20%, respectively, compared to the native host strain lysis effectiveness. Several mesophilic gram‐negative enterobacteria strains were also lysed by AmiP albeit with lower effectiveness (21%) as observed for *Salmonella enterica*. The lytic activity was approximately 12% when *Escherichia coli* or *Pseudomonas fluorescens* strains were used for AmiP substrate specificity evaluation. While no statistically significant activity was observed for other mesophilic gram‐positive strains, *Bacillus subtilis* and *Bacillus pumilus* strains were lysed by AmiP with lytic activity reaching 32% and 15%, respectively. These results demonstrate a wide range of target substrates for AmiP.

**TABLE 1:** AmiP substrate specificity

	Strain	Relative lytic activity (%)
Gram‐negative bacteria	*Thermus parvatiensis* (RL) DSM 21745	100.0 ± 1.0
	*Thermus thermophilus* (HB8) DSM 579^T^	89.3 ± 6.9
	*Thermus flavus* MAT 1087	105.4 ± 0.7
	*Thermus scotoductus* MAT 2631	81.2 ± 1.8
	*Escherichia coli* (K12) DSM 18039	12.7 ± 1.1
	*Pseudomonas fluorescens* DSM 50090^T^	12.3 ± 2.3
	*Salmonella enterica* subsp. *enterica* ser. Panama KPD 101	21.0 ± 4.7
Gram‐positive bacteria	*Bacillus megaterium* DSM 32^T^	ND
	*Bacillus cereus* DSM 6127	ND
	*Bacillus mycoides* KPD 15	ND
	*Bacillus pumilus* KPD 181	14.6 ± 3.0
	*Bacillus subtilis* subsp. *spizizenii* DSM 347	31.6 ± 8.5
	*Bacillus thuringiensis* KPD 114	ND
	*Clostridium sporogenes* DSM 767	1.3 ± 0.9
	*Micrococcus luteus* KPD 104	0.7 ± 0.8
	*Staphylococcus aureus* DSM 1104	ND
	*Staphylococcus epidermidis* DSM 1798	ND
	*Staphylococcus intermedius* KPD 105	ND

*Note*: Relative activities, expressed as the percentage of *Thermus parvatiensis* DSM 21745 activity. Values represent the mean ± standard deviation (*n* = 3). ND, lytic activity not detected.

### 2.7 AmiP structure determination

The crystal structure of AmiP was determined to unravel the molecular basis of thermoactivity and thermostability as well as substrate specificity. Diffraction data were collected to a resolution of 1.79 Å and the structure was refined to a final *R*_work_ 0.185 and *R*_free_ 0.225. Crystallographic data are summarized in Table 2. The structure solution by single‐wavelength anomalous diffraction revealed five independent AmiP molecules in the crystallographic asymmetric unit. Each of the molecules was almost complete with only a few residues missing at the N‐ and C‐terminus including the N‐terminal His‐tag, which were disordered and hence not included in the structure model. The five independent AmiP molecules comprise of residues 7–176 (A), 6–175 (B), 9–176 (C), 8–175 (D) and 8–175 (E), respectively. Each molecule contains also a fully occupied Zn^2+^ ion. All protein chains adopt the same conformation with RMSD values for least‐squares superpositions between 0.20 and 0.22 Å, respectively. The orientation of the five molecules is most likely due to crystal packing as all protein–protein interfaces cover only a surface of approximately 600 Å^2^, a value typically too small for stable dimer formation in solution. The structure model also included five HEPES and five glycerol molecules, five sulfate, two sodium and two chloride ions originating from crystallization conditions as well as 251 water molecules.

**TABLE 2:** Crystallographic data

	AmiP (PDB 7B3N)
*Data collection*	
Beamline	I03, DLS
Space group	P2_1_2_1_2_1_
*Unit‐cell parameters*	
a (Å)	64.54
b (Å)	97.99
c (Å)	148.72
Wavelength (Å)	1.2824
Resolution (Å)	46.53–1.79 (1.83–1.79)
No. of observations	86,433 (3209)
*R*_merge_	0.035 (1.132)
*R*_p.i.m._	0.035 (1.132)
<*I*/*σ*(*I*)>	9.2 (0.8)
CC_1/2_	0.999 (0.283)
Completeness (%)	96.8 (69.9)
Multiplicity	1.9 (1.9)
Anomalous completeness (%)	96.5 (69.3)
*Refinement*	
*R*_work_/*R*_free_	0.185/0.225
No. of atoms	12,680
Ligands	5 Zn^2+^, 5 HEPES, 5 glycerol, 5 sulfates, 2 Na^+^, 2 Cl^−^
No. of waters	251
R.m.s.d. bonds (Å)	0.0103
R.m.s.d. angles (°)	1.642
Ramachandran plot	
Favored (%)	98.80
Allowed (%)	0.60

*Note*: Values in parentheses refer to the highest resolution shell.

### 2.8 AmiP structure and catalytic site architecture

**FIGURE 5:**
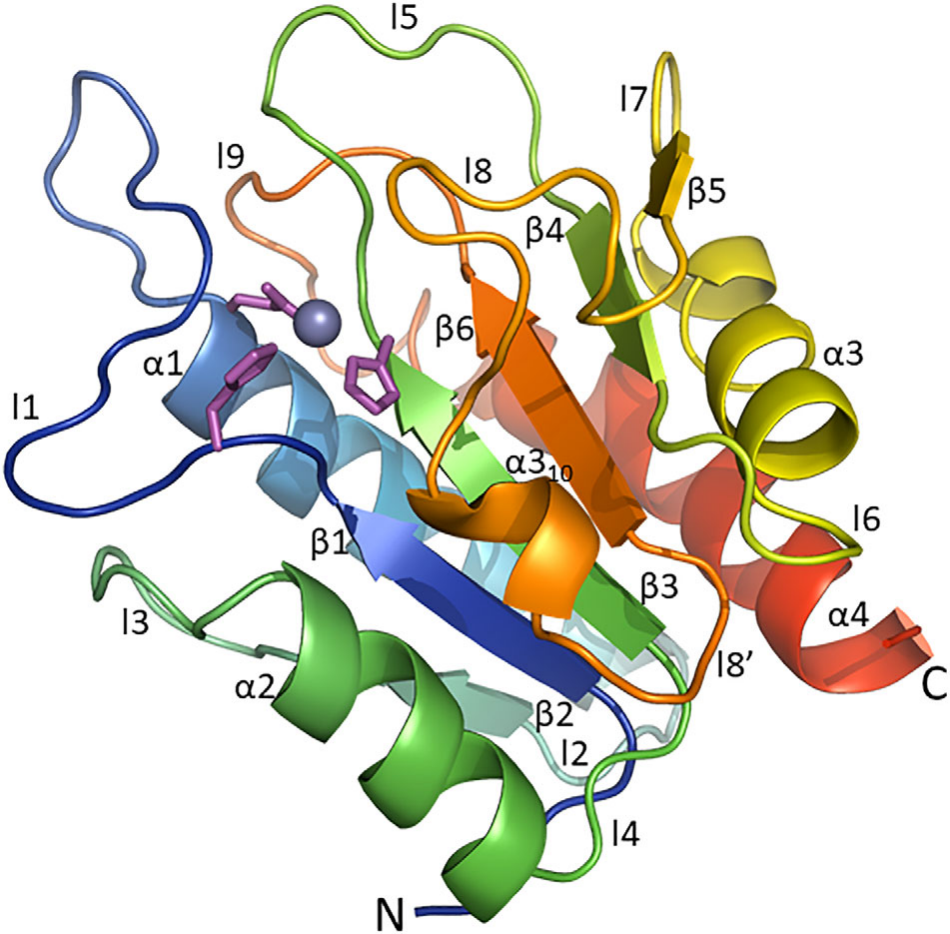
The overall structural organization of AmiP amidase. Ribbon diagram of AmiP with secondary structure elements assigned in rainbow coloring from N‐terminus (blue) to C‐terminus (red) and Zn^2+^ ion coordination in purple coloring in stick representation

**FIGURE 6:**
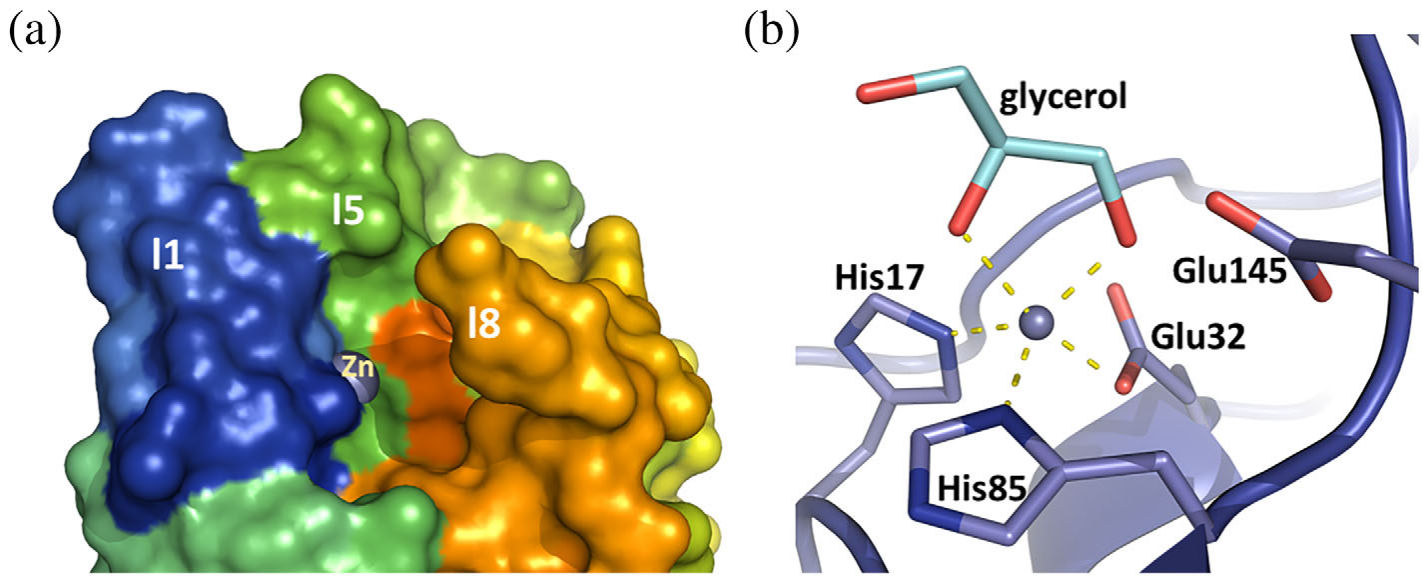
The active site location and the catalytic site architecture of AmiP amidase. (a) AmiP active site surface representation (colors correspond to rainbow coloring) depicting loop l8 bending over the binding site cavity were the Zn^2+^ ion (purple sphere) is deep buried. (b) Zn^2+^ ion coordination in AmiP catalytic site by His^17^, His^85^, Glu^32^, and glycerol molecule with Glu^145^ acting as catalytic residue in stick representation

The overall structural organization of AmiP adopts an Amidase_3 domain fold as described for lytic amidases (Firczuk & Bochtler, 2007). The domain consists of a mixed α/β fold with a central six‐stranded β‐sheet surrounded by four α‐helices and nine loops (Figure S5). The central twisted β‐sheet has a strand order β2‐β1‐β3‐β6‐β4‐β5 with strands β4 and β5 being antiparallel to the first four strands. Helices α1, α3 and C‐terminal helix α4 shield the rear side of the β‐sheet, while helix α2 partly covers the β‐sheet front side (Figure 5). An additional 3_10_‐helix in loop l8 also covers the β‐sheet front side. The active site is located in the shallow cavity of the upper end of an extensive recessed cleft defined by loops l1, l5, l8, and helix α2 across the β‐sheet (Figure 6a).

The Zn^2+^ ion in the catalytic site is coordinated by His^17^N^ε2^ (AmiP (B) 2.18 Å), His^85^N^δ1^ (AmiP (B) 2.15 Å) and Glu^32^O^ε2^ (AmiP (B) 2.06 Å). One glycerol molecule (GOL) originating from the crystallization conditions serves as a bidentate ligand completing the Zn^2+^ ion distorted trigonal bipyramidal coordination geometry (Figure 6b). The glycerol molecules are partially disordered and in molecules A and C two positions were refined. The glycerol coordination at the Zn^2+^ ion is in all five molecules different and can be described as distorted tetrahedral in molecule E but is in all other molecules closer to a distorted trigonal bipyramidal arrangement. Observed distances and geometries were consistent with Zn^2+^ ion coordination in proteins (Cornish et al., 2022; Laitaoja et al., 2013). Glu^145^ acting as catalytic residue located in strand β6 is oriented in proximity to the coordinated Zn^2+^ ion. Therefore, the catalytic site architecture is similar to the open active site's characteristic for Amidase_3 domains (Büttner et al., 2015).

### 2.9 AmiP structure similarity and conservation

**FIGURE 7:**
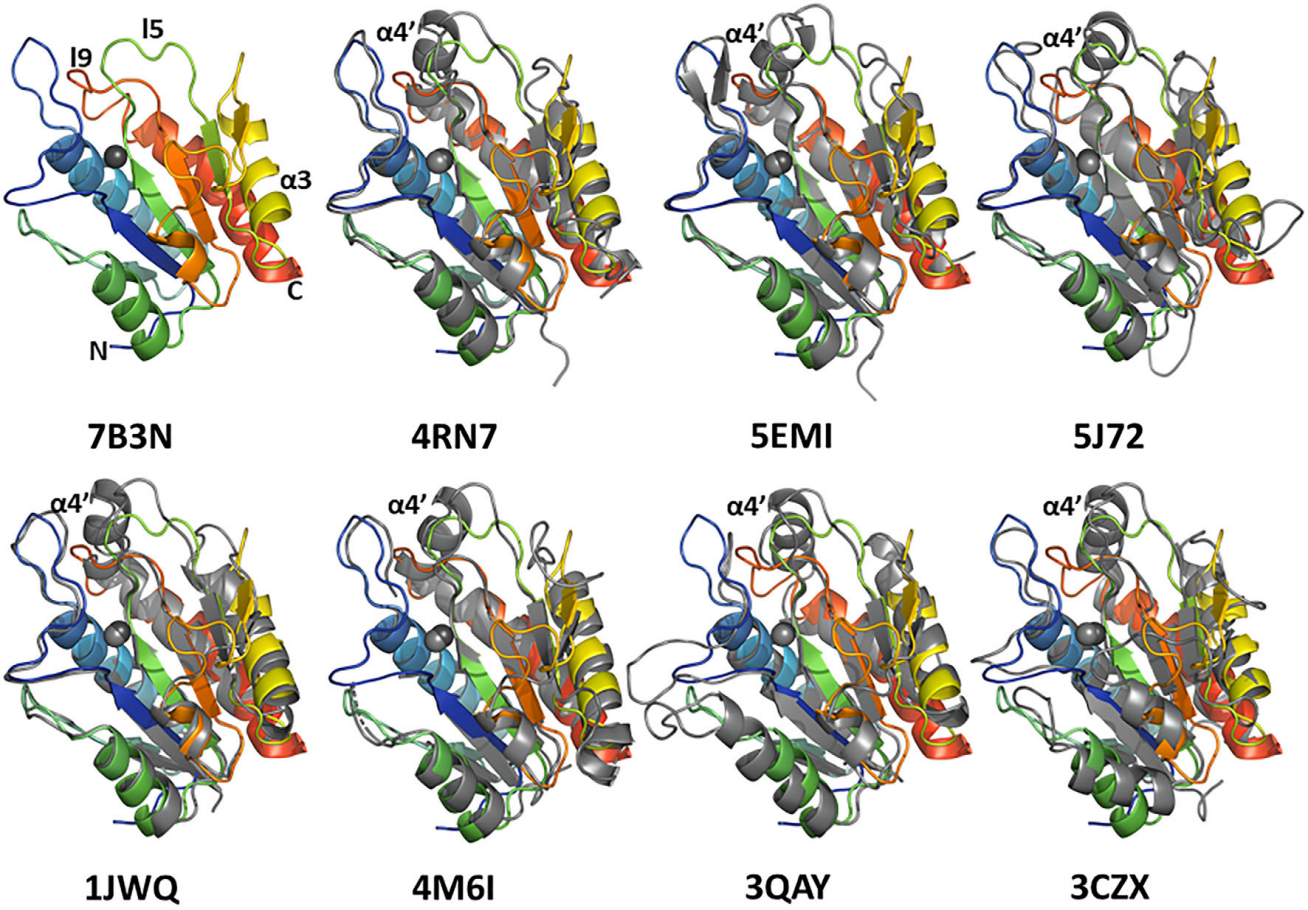
Least‐squares superpositions of AmiP with structural homologues. Ribbon diagram of the AmiP structure (PDB 7B3N) in rainbow coloring from N‐terminus (blue) to C‐terminus (red) superimposed with homologues in gray coloring as ribbon diagram representation. PDB 4RN7 (Tan et al., n.d.)—catalytic domain of putative N‐acetylmuramoyl‐l‐alanine amidase from *Clostridioides difficile*, PDB 5EMI (Büttner et al., 2016)—catalytic domain of AmiC2 cell wall amidase from *Nostoc punctiforme*, PDB 5J72 (Usenik et al., 2017)—amidase domain of Cwp6 cell wall protein from *Clostridioides difficile*, PDB 1JWQ (Yamane et al., 2003)—catalytic domain of CwlV cell wall amidase from *Paenibacillus polymyxa* subsp. *colistinus*, PDB 4M6I (Prigozhin et al., 2013)—Rv3717 peptidoglycan amidase from *Mycobacterium tuberculosis*, PDB 3QAY (Mayer et al., 2011)—catalytic domain of endolysin CD27L from *Clostridioides difficile* phage ϕCD27, PDB 3CZX (Zhang et al., n.d.)—putative N‐acetylmuramoyl‐l‐alanine amidase from *Neisseria meningitidis*. Several SSEs are shorter or absent in AmiP: all β‐sheets, helices α3 and α4′ in loop 9, as well as loop 5.

The least‐squares superpositions of the AmiP structure with homologous lytic Amidase_3 catalytic domains (Figure 7) confirm the high fold and topology similarity with RMSDs between 1.21 and 2.02 Å for 139–156 residues (Table 3). The catalytic domains of putative N‐acetylmuramoyl‐L‐alanine amidase from *Clostridioides difficile* (Tan et al., n.d.) and AmiC2 cell wall amidase from *Nostoc punctiforme* (Büttner et al., 2016) are the most similar to the AmiP structure. The catalytic domains of the cell wall amidase CwlV from *Paenibacillus polymyxa* subsp. *colistinus* (Yamane et al., 2003), peptidoglycan amidase Rv3717 from *Mycobacterium tuberculosis* (Prigozhin et al., 2013), the cell wall amidase Cwp6 from *Clostridioides difficile* (Usenik et al., 2017) as well as the catalytic domain of endolysin CD27L from *Clostridioides difficile* phage ϕCD27 (Mayer et al., 2011) superimposed well with the AmiP structure with RMSDs between 1.41 and 1.57 Å for 139–155 residues. The putative N‐acetylmuramoyl‐l‐alanine amidase from *Neisseria meningitidis* (Zhang et al., n.d.) was the least similar to the AmiP structure among related lytic amidases. Sequence identities between 21% and 34% were observed aligning AmiP with the compared lytic amidases. While the sequence lengths of the structural homologues varied between 179 and 214 residues, AmiP consists of a shorter Amidase_3 domain with only 168 residues. The structure fold and topology of AmiP are less similar to the catalytic domains of the two compared cell separation amidases even though the sequence identities are higher compared with the sequence identities observed aligning the AmiP sequence with lytic amidases sequences. The catalytic domains of cell separation amidases AmiB from *Bartonella henselae* (Yang et al., 2012) and AmiC from *E. coli* (Rocaboy et al., 2013) superimposed with AmiP structure with RMSD 1.28 and 1.55 Å for 150 and 152 residues, respectively.

**TABLE 3:** Comparison of AmiP structure with homologous amidases

PDB	Group	Chain	Name	Residues	Residue number	Sequence identity (%)	Secondary structure element identity (%)	Secondary structure element R.m.s.d. (Å), residues
7B3N	Lytic amidases	A–E	AmiP	8–175	168	100	100	0.22, 168
4RN7 (Tan et al., n.d.)		A	Amidase	114–299	186	29	83	1.21, 156
5EMI (Büttner et al., 2016)		A	AmiC2	435–614	180	34	77	1.29, 151
5J72 (Usenik et al., 2017)		A–B	Cwp6	457–647	191	28	71	1.50, 155
1JWQ (Yamane et al., 2003)		A	CwlV	1–179	179	29	83	1.41, 153
4M6I (Prigozhin et al., 2013)		A–B	Rv3717	24–237	214	29–30	75	1.50, 150
3QAY (Mayer et al., 2011)		A–D	CD27L	1–180	180	21	91	1.57, 139
3CZX (Zhang et al., n.d.)		A–D	Amidase	1–182	182	23–24	73–82	2.02, 144
3NE8 (Yang et al., 2012)	Cell separation amidases	A	AmiB	178–409	232	39	58	1.28, 150
4BIN (Rocaboy et al., 2013)		A	AmiC	175–408	234	44	47	1.55, 152

For the comparison of the characteristic features of the AmiP secondary structure with related lytic amidases the structures were superimposed (Figure 7) revealing omission and shortening of several secondary structure elements (Table S2). In contrast to other lytic amidases, AmiP features only four helices instead of five as a short helix α4′ before the C‐terminal helix α4, which is conserved in all compared amidases, is not present in AmiP. In addition, helix α3 as well as all strands and loop l5 are shorter in AmiP compared to the other lytic amidase structures. The overall structure of AmiP represents the most compact Amidase_3 domain of all structurally related lytic amidases.

### 2.10 AmiP interactions with substrate

**FIGURE 8:**
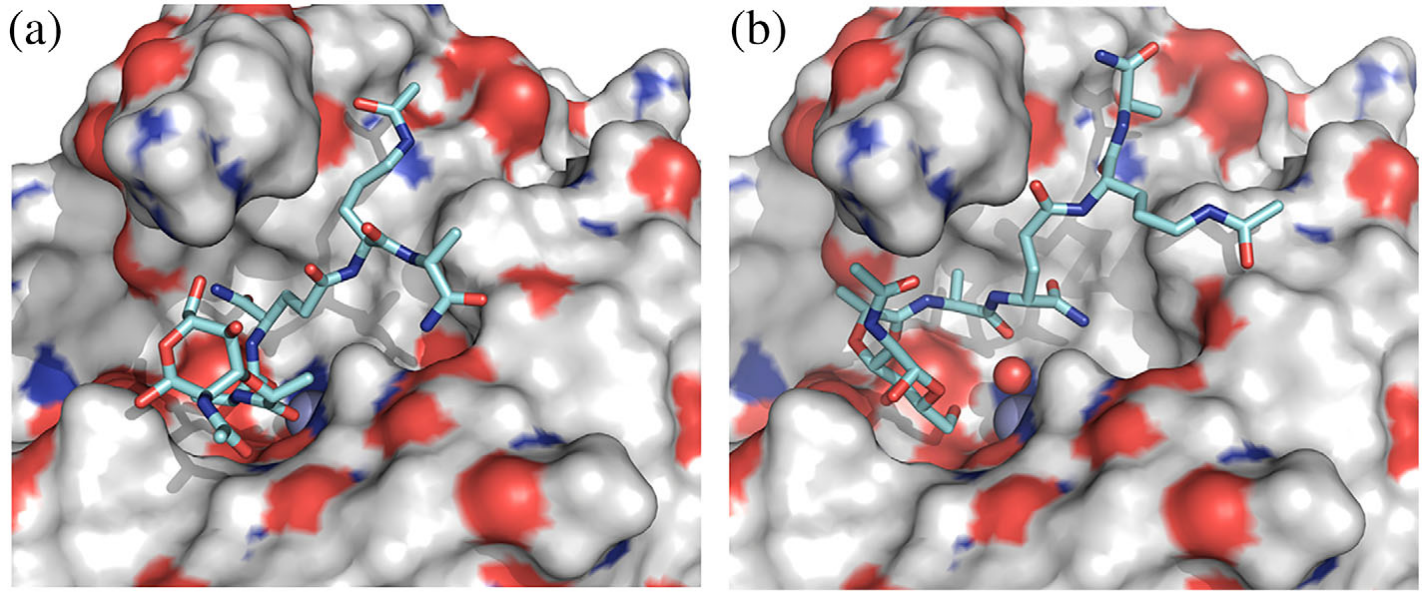
AmiP interactions with MTP MurNAc‐l‐Ala‐d‐iGln‐l‐Orn‐NHAc‐d‐Ala‐NH_2_ accommodated in the active site were calculated with GOLD software (Jones et al., 1995). The Connolly surface representation of AmiP (colors correspond to atom types) depicting the active site substrate binding pocket with Zn^2+^ ion (gray sphere) deep buried. (a) The muramyl tetrapeptide highest‐ranked docking pose. (b) The muramyl tetrapeptide highest‐ranked docking pose including the water molecule (red sphere)

Docking calculations of AmiP with a potential substrate, a muramyl tetrapeptide MurNAc‐l‐Ala–D‐iGln‐l‐Orn–NHAc‐d‐Ala‐NH_2_ (MTP) were performed to further investigate the reaction mechanism. As hydrolysis involves an activated water molecule interacting with the Zn^2+^ ion in a transition state, a water molecule was introduced, replacing one of the glycerol oxygen atoms of the glycerol molecule identified in the AmiP crystal structure. The flexible docking algorithm in the GOLD software (Jones et al., 1995) allows excluding the water molecule if energetically favorable. The top scored 30 docking poses of MTP in the AmiP active site (Table S3) are, as expected for a large and flexible ligand, highly diverse, but in all binding poses the MTP integrated very well into the AmiP binding grove. It is noticeable that the best scoring binding poses do not include the water molecule in the binding site. The highest‐ranked docking pose (Figure 8a) shows Zn^2+^ ion interactions to the l‐Ala carbonyl oxygen atom (2.3 Å), as well as interactions of the Zn^2+^ ion to the MurNAc carbonyl oxygen atom (2.2 Å), which is exactly at the catalytically targeted peptide bond. Three hydrogen bonds are formed upon muramyl tetrapeptide docking between l‐Ala‐CO‐Glu^145^O^ε2^ (2.5 Å), d‐Ala‐CO‐Tyr^63^OH (2.8 Å) and d‐Ala‐NH_2_‐Asp^21^O^δ2^ (3.0 Å; Figure S6). The overall AmiP binding pocket size and sufficient ligand flexibility allows the positioning of an activated water molecule between the Zn^2+^ ion and the catalytically targeted peptide bond (Figure 8b). Therefore, the predicted reaction mechanism via a nucleophilic attack by an activated water molecule implied for AmiP was confirmed.

## 3 DISCUSSION

Cell wall amidases together with peptidoglycan glycosidases and peptidoglycan peptidases ensure peptidoglycan disruption critical for the phage life cycle (Vermassen et al., 2019). Considerable progress was made characterizing peptidoglycan amidases, however amidases containing Amidase_3 type catalytic domains from bacteriophages infecting thermophilic bacteria or encoded in prophage sequences identified in genomes of thermophilic bacteria remain sporadically investigated (Aevarsson et al., 2021; Fernández‐Ruiz et al., 2018). The detailed characterization of AmiP presented here reveals main characteristics of a thermoactive and ultrathermostable Amidase_3 as well as its adaptation traits to high temperatures.

Homology search and sequence analysis of AmiP confirms close evolutionary relationships with peptidoglycan amidases encoded in *Thermus* and *Meiothermus* prophage sequences as well as encoded by phages infecting *Thermus* strains. *Deinococcus* strains also encode amidases evolutionarily related to AmiP amidase with significant sequence identity. Comparatively low sequence identity and distant yet traceable evolutionary relationships are determined between AmiP, and structural homologues encoded by mesophilic microorganisms. AmiP transportation through the cell membrane is most likely ensured by prophage encoded holins (Jin et al., 2013; Vermassen et al., 2019) as a signal peptide or transmembrane regions are not predicted in the AmiP sequence. The sequence analysis of the catalytic domain of AmiP corresponds to residue frequencies characteristic for proteins from thermophilic and hyperthermophilic microorganisms (Hait et al., 2020; Taylor & Vaisman, 2010; Walden et al., 2004). Thermolabile residues such as Asn and Gln undergoing deamidation as well as Met and Cys prone to oxidation appear less frequent in AmiP's catalytic domain compared to related amidases from mesophilic organisms. Arg and Pro are less frequent in lytic amidases catalytic domains from mesophilic organisms but are comparatively overrepresented in the catalytic domain of AmiP as typical for a thermostable protein. Arg facilitates intramolecular cation‐π interactions, while the abundance of Pro contributes to the structural compactness of thermoactive and thermostable proteins (Hait et al., 2020). The overall amino acid composition of the AmiP enzyme presented here confirms the adaptation to high temperatures.

AmiP is the most thermoactive and thermostable lytic amidase of all biochemically characterized Amidase_3 type lytic amidases with a temperature optimum at 85°C. It shows residual lytic activity between 90% and 98% after preincubation for 30 min at temperatures of 60–99°C. The amidase withstands even autoclavation for 20 min at 121°C and shows relative residual lytic activity of 84% after autoclavation. AmiP remains active after preincubation for 0.5–6 h at 99°C, only losing activity completely after preincubation for 13 h at 99°C. The amidase demonstrates fold stability up to approximately 100°C as thermal unfolding was not observed between 20 and 100°C, while the aggregation onset temperature is approximately 100°C. The extraordinary high melting temperature *T*_m_ 102.6°C and mid‐aggregation temperature *T*_agg_ 102.0°C characterizes AmiP as a remarkably ultrathermostable protein. Amidase_3 type amidases from mesophilic bacteria or phages infecting mesophiles are not thermoactive and withstand only temperatures lower than 50°C (Mehta et al., 2013). Moreover, thermoactivity and thermostability of orthologues encoded by thermophilic microorganisms or phages as well as fusion enzymes containing Amidase_3 catalytic domain from thermophilic bacteria or phages do not exceed unfolding temperatures above 70°C (Swift et al., 2019). AmiP is significantly more thermoactive and thermostable than GVE2 lysin, which is currently considered the most thermostable Amidase_3 type lytic enzyme (activity optimum at 60–70°C, thermostable at 50–60°C for at least 1.5 h; Ye & Zhang, 2008). AmiP's thermostability can only be compared with the phage endolysins Ph2119 (Plotka et al., 2014) and Ts2631 (Plotka et al., 2015; Plotka et al., 2019) attributed to Amidase_2 type from phages infecting *Thermus scotoductus* strains. However, none of these amidases remain active after autoclavation. AmiP demonstrates also high efficiency of catalytic activity at 60°C. The lysis reaction saturation was reached for the native host *T. parvatiensis* strain at 1 μg/mL concentration after 9 min incubation. Amidase_3 type lytic enzymes from mesophilic bacteria are comparatively less efficient in disrupting the peptidoglycan (Kumar et al., 2013; Mehta et al., 2013). Amidase_3 type amidases are typically active over a broad pH range with pH optimum varying between pH 6.0 and 8.5 (Swift et al., 2019). AmiP was more active over the alkaline pH range with a pH optimum at pH 8.5. The optimal NaCl concentration for AmiP activity was established at 25 mM. NaCl concentrations up to 300 mM were well‐tolerated by AmiP, however NaCl concentrations above 400 mM decreased AmiP activity. This corresponds to NaCl tolerance ranges and optimal NaCl concentrations characteristic for this enzyme family.

The antimicrobial activity of AmiP was demonstrated toward a range of thermophilic and mesophilic bacteria strains containing Orn‐type or DAP‐type peptidoglycan. *Thermus* strains were lysed by AmiP most effectively in comparison with selected *Escherichia*, *Pseudomonas*, and *Salmonella* strains, what confirms AmiP as the most effective in disrupting Orn‐type peptidoglycan with L‐Orn at the third position of the stem peptide that is typical for gram‐negative thermophilic bacteria. *B. subtilis* and *B. pumilus* strains were also lysed by AmiP. Similar to most gram‐negative bacteria, such as *Escherichia*, *Pseudomonas*, and *Salmonella* strains, *Bacillus* strains contain peptidoglycan with meso‐diaminopimelic acid (DAP) at the third position of the stem peptide. This confirms AmiP's ability to disrupt DAP‐type peptidoglycan. However, AmiP was not able to disrupt Lys‐type peptidoglycan with l‐Lys at the third position of the stem peptide that is typical for gram‐positive bacteria such as *Staphylococcus aureus* or *Micrococcus luteus*. The substrate specificity of AmiP was influenced not only by peptidoglycan type but also by secondary substrate modifications as only *B. subtilis* and *B. pumilus* gram‐positive strains were lysed by this amidase. AmiP lacks the cell wall binding domain (CBD) governing peptidoglycan binding specificity ensuring a broad substrate specificity typical for globular endolysins from phages infecting gram‐negative bacteria (Vermassen et al., 2019).

The overall AmiP structure reveals an Amidase_3 domain fold and topology with accessible open active site. The structural organization of AmiP represents an Amidase_3 catalytic domain of the shortest sequence ever characterized. Typical Amidase_3 domains have an estimated length of 175 residues (Vermassen et al., 2019), while AmiP consists of only 168 residues. The Amidase_3 domain sequence length of AmiP structural homologues varied between 179 and 214 residues. Compared to these structures AmiP adopts a distinct compact organization achieved by omission of a helix α4′ and shortening of helix α3 as well as all β‐strands and loop l5. The short helix α4′ which is not present in AmiP's secondary structure is conserved in all AmiP structural homologues compared here. AmiP's compactness confirms a trait associated with adaptation to high temperatures as it was shown before that hyperthermophilic proteins are usually shorter than mesophilic homologues (Walden et al., 2004). Intramolecular activity regulatory elements typical for cell separation amidases (Do et al., 2020) and also characterized in structures of lytic amidases are absent. Docking calculations of AmiP with MTP selected as a potential substrate confirm the peptide bond breakage mechanism via a nucleophilic attack by an activated water molecule on the Zn^2+^ ion in the catalytic site. MTP is well‐accommodated into the substrate binding pocket as well as into the substrate binding grove. These molecular modeling studies predict direct coordination of the Zn^2+^ ion in the catalytic center of AmiP by the MTP ligand through two hydrogen bonds without a water molecule in the substrate binding pocket. Direct Zn^2+^ ion coordination by the substrate ligand was also determined in the crystal structure of the Amidase_2 type autolysin AtlA complex with a muramyl tetrapeptide bound (*S. aureus* AmiA, PDB 4KNL; Büttner et al., 2014). However, the overall AmiP binding pocket size and sufficient ligand flexibility allow the positioning of an activated water molecule between the Zn^2+^ ion and the catalytically targeted peptide bond. The docking results of MTP into the AmiP structure explain a broad substrate specificity similar to structurally related lytic amidases disrupting Orn‐type and DAP‐type peptidoglycans (Swift et al., 2019). The active site location in the shallow cavity of an extensive recessed cleft also confirms AmiP's active site accessibility and thus the broad substrate specificity demonstrated in our lytic assays.

Our studies show that AmiP is active under a wide range of conditions (0–100°C, pH 6.5–10.0, 0–300 mM NaCl). The optimal conditions are 85°C, pH 8.5 and 25 mM NaCl concentration. This is in agreement with the unfolding and aggregation temperatures at 102.6 and 102.0°C, respectively. AmiP is most effective toward gram‐negative *Thermus* and Salmonella strains, but also some gram‐positive *Bacillus* (*B. pumilus* and *B. subtilis*) strains. The docking studies of AmiP and the potential target MTP also underline a relatively extensive target pool. Sequence and structural characterization dissected the basis of AmiP's adaption to very high temperatures. This is first defined by the compactness of the AmiP 3D‐structure and secondly by the absence of thermolabile and presence of thermostable amino acids in its overall short sequence. The straightforward heterologous expression and purification allows a relatively convenient production of stable and non‐aggregation prone amidase for a wide range of applications. In conclusion, AmiP from *T. parvatiensis* DSM 21745 prophage constitutes an exceptional potent enzyme with a set of beneficial characteristics, which could be in the future exploited in biotechnology or implemented in clinical applications.

## 4 MATERIALS AND METHODS

### 4.1 Gene identification and sequence analysis

The prophage sequences in bacterial genomes were localized with PHASTER (Arndt et al., 2016). All ORF predictions in prophage sequences were refined with the NCBI BLAST suite. The functional annotation of prophages protein encoding genes was performed with InterProScan (Jones et al., 2014) based on the Pfam database (El‐Gebali et al., 2019) and NCBI CDD (Lu et al., 2020). Protein sequences were compared with blastp implemented in the NCBI BLAST (Altschul et al., 1990) suite when necessary, choosing nr, swissprot or pdb sequence databases. Multiple sequence alignments were generated with the 3DM protein superfamily data analysis software suite (Bio‐Prodict BV) under default parameter values (Kuipers et al., 2010) aligning AmiP with all homologous protein sequences accessible via UniProtKB (>37,000 sequences). The generated Amidase_3 superfamily alignment was further developed with the 3DM suite by grouping protein sequences in subfamilies based on integrated AmiP structure comparison with Amidase_3 domain structures. AmiP subfamily representatives clustered at 95% (425 sequences) were extracted excluding non‐aligned domain sequence regions. Extracted sequences were re‐aligned with ClustalW implemented in the MEGA X suite (Kumar et al., 2018) under default parameter values for AmiP phylogenetic analysis. MEGA X suite was also used to calculate maximum‐likelihood (Jones et al., 1992) trees under default parameter values with 500 bootstrap replications (Felsenstein, 1985). Multiple sequence alignments and phylogenetic relationships with structurally characterized homologues analysis were prepared analogously. The graphical representations of phylogenetic trees were created with iTOL (Letunic & Bork, 2019). Amino acid composition analysis of Amidase_3 domain sequences retrieved from UniProtKB was performed with ExPaSy ProtParam tool (Gasteiger et al., 2003).

### 4.2 Bacteria strains and cultivation

The strains used were obtained from the German Collection of Microorganisms and Cell Cultures (DSMZ, Braunschweig, Germany), the Collection of Plasmids and Microorganisms (KPD, Gdansk, Poland), and Matís ltd. (MAT, Reykjavik, Iceland). *Bacillus*, *Escherichia*, *Micrococcus*, *Salmonella*, and *Staphylococcus* strains were propagated from culture freezer stocks aerobically on LB‐Lennox agar or in LB‐Lennox medium at 37°C. *Thermus* strains were cultivated aerobically in TM medium (Plotka et al., 2014) at 60°C. The *Clostridium* strain was cultivated anaerobically in a TSB medium at 37°C.

### 4.3 Cloning, protein production, and purification

The full‐length coding region of the *amiP* gene was amplified by PCR from *T*. *parvatiensis* DSM 21745 genomic DNA (DSMZ, Braunschweig, Germany) using gene‐specific primers s12329: 5′‐aaaaaagatctGTGCGCCGCCAAGCGC‐3′ and s12097: 5′‐aaaaaaagcttACGCCGTCCACCTCCTC‐3′ with *Bgl*II and *Hin*dIII restriction sites underlined, respectively. The *amiP* gene including a sequence encoding the N‐terminal His_6_‐tag was incorporated into the rhamnose‐inducible vector pJOE5751.1 (Wegerer et al., 2008) via *Bam*HI and *Hin*dIII restriction sites. The expression construct verified by sequencing was transformed into *E. coli* JM109 (Promega). Expression culture was cultivated in LB‐Lennox medium. Heterologous production of AmiP was induced at OD_600_ 0.3–0.4 of expression culture with 0.2% (wt/vol) l‐rhamnose for 4 h at 30°C. Cells were subsequently harvested by centrifugation and lysed by ultra‐sonication using UP400S homogenizer (Hielscher Ultrasonics). The lysate was separated from cell debris by centrifugation 26,000*g* for 30 min at 4°C. Then, the supernatant was filtered through regenerated cellulose 0.2 μm pore size filters (GE Healthcare Life Sciences).

AmiP was purified by nickel affinity chromatography using HisTrap HP 1 mL (7 × 25 mm) columns (GE Healthcare Life Sciences) with the ÄKTA Start FPLC purification system (GE Healthcare Life Sciences). The His‐tagged protein bound to resin in lysis buffer (50 mM Tris–HCl pH 7.4, 60 mM imidazole, 10% (vol/vol) glycerol, 500 mM NaCl) and was eluted after extensive washing with lysis buffer (8 CV) with elution buffer (50 mM Tris–HCl pH 7.4, 500 mM imidazole, 10% [vol/vol] glycerol, 500 mM NaCl). The protein was dialysed into a reaction buffer (25 mM K_2_HPO_4_‐KH_2_PO_4_ pH 8.0, 0.2 mM ZnSO_4_, 60% [vol/vol] glycerol, 50 mM KCl, 10 mM β‐mercaptoethanol, 0.1% [vol/vol] Triton X‐100). AmiP identity, integrity and purity were assessed by electrospray ionization mass spectrometry and 4%–15% glycine‐SDS‐PAGE. The protein concentration was determined considering the absorption coefficient by measuring A_280_ using a NanoDrop 1000 spectrophotometer (Thermo Scientific).

### 4.4 Lytic activity measurement

The activity of AmiP was assayed spectrophotometrically by measuring turbidity reduction of the cell substrate suspension. The turbidity reduction assay was performed in a 96‐well plate format with chloroform treated *T. thermophilus* (HB8) DSM 579^T^ substrate used for dose range and biochemical activity characterization as well as *T. parvatiensis* (RL) DSM 21745 substrate also used for dose range characterization. The substrates were prepared from strain cultures harvested at the late logarithmic growth phase (Lavigne et al., 2004). The assay reaction contained 190 μL of the substrate and 10 μL of AmiP solution diluted to achieve a final concentration in the range of 0.0625–25.0 μg/mL. The substrate suspensions were adjusted to OD_600_ 1.0 with initial reaction buffer (10 mM K_2_HPO_4_‐KH_2_PO_4_, pH 8.0) and subsequently with optimal reaction buffer (25 mM Tris–HCl pH 8.5, 25 mM NaCl). AmiP solution dialysed against reaction buffer was used for dilutions. The assay reaction was incubated at the indicated temperature measuring OD_600_ over time using an EnSpire multimode plate reader (PerkinElmer). Negative control reactions contained reaction buffer instead of enzyme solution. The lytic activity was calculated by subtracting the negative control ΔOD_600_ value from the measured ΔOD_600_ values after indicated reaction incubation time divided by initial OD_600_ value:

$$\frac{\mathrm{\Delta OD}600\;\text{sample}\;\left(\text{enzyme solution}\right)-\mathrm{\Delta OD}600\;\text{negative control}\;\left(\text{only reaction buffer}\right)}{\text{initial}\;\mathrm{OD}600}$$

All measurements were performed in triplicates.

### 4.5 Activity optimum characterization

The pH optimum of AmiP was determined over a pH range 5.0–10.0 in buffers (25 mM CH_3_COONa‐CH_3_COOH, pH 5.0 and 5.5, 25 mM MES‐NaOH, pH 6.0, 25 mM K_2_HPO_4_‐KH_2_PO_4_, pH 6.5, 7.0 and 7.5, 25 mM Tris–HCl, pH 8.0 and 8.5, 25 mM glycine‐NaOH, pH 9.0 and 9.5 as well as 25 mM CAPS‐NaOH, pH 10.0) measuring the lytic activity after incubation for 20 min at 60°C. The effect of NaCl concentration on activity was determined by performing the turbidity reduction assay in a reaction mixture containing 1 μg/mL of enzyme in optimal pH buffer (25 mM Tris–HCl, pH 8.5) supplemented with NaCl in a range of 0–750 mM. The thermoactivity and thermostability of AmiP were determined by measuring residual lytic activity at 60°C performing the turbidity reduction assay. Preincubation of 0.2 μg enzyme aliquots in optimal reaction buffer was performed for 30 min at 0–99°C and 20 min at 121°C (autoclavation at 1 atm) or 0.5–13 h at 99°C prior for thermostability determination. AmiP aliquots were cooled on ice after preincubation for 5 min.

The substrate specificity of AmiP was evaluated by performing a turbidity reduction assay with substrates prepared from selected bacterial cultures harvested at the late logarithmic growth phase. Gram‐negative bacteria cells were chloroform treated (Lavigne et al., 2004) while gram‐positive bacteria cells were prepared by extensive washing with optimal reaction buffer. Turbidity reduction assay reactions were incubated for 20 min at 37 or 60°C before performing the assay with substrates from mesophilic or thermophilic bacteria, respectively.

### 4.6 Thermostability and thermoaggregation characterization

Thermostability and thermoaggregation of AmiP were assayed by nanoscale differential scanning fluorimetry and backreflection technology estimating thermal unfolding and light scattering, respectively. Measurements were performed with Prometheus NT.48 instrument (NanoTemper Technologies) processing results with PR.ThermControl software (NanoTemper Technologies). Thermostability and thermoaggregation of AmiP at 2 mg/mL concentration in optimal reaction buffer supplemented with 5% (vol/vol) glycerol were assayed by performing measurements using standard grade capillaries (NanoTemper Technologies). AmiP melting temperature (*T*_m_ 103°C) was determined by thermal unfolding at 20% excitation power with a temperature gradient between 20°C and 110°C at a ramp rate of 1°C/min. Thermal unfolding was measured by tryptophan and tyrosine fluorescence change at 330 and 350 nm emission wavelengths. Simultaneously, AmiP mid‐aggregation temperature (*T*_agg_ 102°C) was determined by estimating light scattering by measuring backreflection light intensity change. All measurements were performed in triplicates.

### 4.7 Crystallization, data collection, structure solution, and refinement

Initial AmiP crystallization trials were performed using the Mosquito Crystal robot (SPT Labtech) in a 96 × 2 MRC plate sitting drop format using a range of commercial crystallization screens (Molecular Dimensions). Crystals were obtained using a protein solution containing 5.0 mg/mL of AmiP in 20 mM MES‐NaOH, pH 6.0 buffer with a reservoir solution consisting of 100 mM HEPES‐NaOH pH 7.8, 65% (vol/vol) 2‐methyl‐2,4‐pentanediol, at room temperature. AmiP crystallized in the form of rods (∼0.2 × 0.2 × 0.3 mm). Crystals were transferred into cryosolution (reservoir solution and 50% [vol/vol] glycerol solution at a 1:1 ratio) and flash‐cooled in liquid nitrogen before data collection (Garman, 2003; Teng, 1990). Diffraction data were collected using an Eiger2 XE 16 M pixel‐array detector (Broennimann et al., 2006) on beamline I03 at the Diamond Light Source (DLS) facility (Didcot, UK). The structure was solved by single‐wavelength anomalous diffraction with DLS Big_ep advanced experimental phasing pipeline employing xia2 (Winter et al., 2013), autoSHARP (Vonrhein et al., 2007), AutoSol (Terwilliger et al., 2009), CRANK2 (Skubák & Pannu, 2013) and the SHELX program package (Sheldrick, 2010; Usón & Sheldrick, 2018). As the collected data quality deteriorated clearly as documented, for example, by a significant increase in mosaicity, not all data images were being used for re‐processing of the data with XDS (Kabsch, 2010), which resulted in the data set used for the crystallographic refinement of the model with REFMAC5 (Murshudov et al., 2011) implemented in the CCP4 suite (Winn et al., 2011). All model building and evaluation were performed with Coot (Emsley et al., 2010). Model validation was performed with MolProbity (Chen et al., 2015). Crystallographic data are summarized in Table 2.

### 4.8 Structure analysis and comparison

The structural similarity was determined by superimposing the AmiP crystal structure with protein structures and comparing secondary structures. Least‐squares superpositions of Cα‐atoms or secondary structure matching methods were performed with CCP4mg (McNicholas et al., 2011). Structure alignments were also performed with the PDBeFold service (Krissinel & Henrick, 2004). The graphical representations of multiple sequence alignments were created with ESPript3 (Robert & Gouet, 2014).

### 4.9 Docking calculations and interaction solution

AmiP interactions with the potential substrate MTP were evaluated with the GOLD suite (Jones et al., 1995). Hydrogen atoms have been added to the AmiP and substrate structures before docking calculations. One of the glycerol oxygen atoms was included for docking calculations as a Zn^2+^ ion interacting water molecule, while all other ligands in the crystal structure have been omitted. The muramyl tetrapeptide ligand of the structure of bifunctional major autolysin AtlA AmiA catalytic domain from *S. aureus* (PDB 4KNL; Büttner et al., 2016) was the start model for MTP MurNAc‐l‐Ala‐d‐iGln‐l‐Orn–NHAc‐d‐Ala‐NH_2_. The l‐Lys amino acid was altered to an l‐Orn amino acid to accommodate the *Thermus* bacteria peptidoglycan composition. The docking process involved high accuracy settings first with GoldScore and rescoring with ChemScore for 30 genetic algorithm runs, enabling high ligand flexibility (Tatum et al., 2017). The range for GoldScore fitness was −22 to 65 and for ChemScore fitness −46 to −13. All interaction solutions were analyzed after visualization with Hermes implemented in the GOLD suite estimating hydrogen bonds and too close distances. The docking poses with the highest GoldScore and ChemScore fitness did not involve a water molecule in the binding site (Table S3). This could be due to entropic contributions. The docking pose with the highest ChemScore fitness of −13 had the third‐ranked GoldScore fitness of 56 and was interpreted as most probable. The graphical representations of AmiP interaction with MTP were created with PyMOL2 (Schrödinger).

### 4.10 Structure deposition

The final coordinates and structure factors have been deposited in the Protein Data Bank (PDB) with accession code 7B3N (AmiP).

## AUTHOR CONTRIBUTIONS

**Andrius Jasilionis:** Data curation (equal); formal analysis (equal); investigation (equal); writing – original draft (lead). **Magdalena Plotka:** Data curation (equal); formal analysis (equal); investigation (equal); visualization (equal). **Lei Wang:** Data curation (equal); investigation (equal). **Sebastian Dorawa:** Data curation (equal); formal analysis (equal); investigation (equal); visualization (equal). **Joanna Lange:** Data curation (equal); formal analysis (equal); investigation (equal); visualization (equal). **Hildegard Watzlawick:** Data curation (equal); investigation (equal). **Tom van den Bergh:** Data curation (equal); formal analysis (equal); investigation (equal); visualization (equal). **Bas Vroling:** Data curation (equal); formal analysis (equal); investigation (equal); visualization (equal). **Josef Altenbuchner:** Data curation (equal); investigation (equal). **Anna‐Karina Kaczorowska:** Conceptualization (equal); funding acquisition (equal). **Ehmke Pohl:** Conceptualization (equal); funding acquisition (equal). **Tadeusz Kaczorowski:** Conceptualization (equal); funding acquisition (equal). **Eva Nordberg Karlsson:** Conceptualization (equal); funding acquisition (equal). **Stefanie Freitag‐Pohl:** Data curation (equal); formal analysis (equal); investigation (equal); validation (equal); visualization (equal); writing – original draft (equal); writing – review and editing (lead).

## CONFLICT OF INTEREST STATEMENT

The authors declare no conflicts of interest.

## Supporting information


**Data S1:** Supporting information

**Table S1.** Amino acid composition of AmiP and homologous Amidases_3 domains as found in Uniprot.

**Table S2.** Comparison of the secondary structure elements of AmiP with homologous lytic amidases by least squares superposition and subsequent visual assessment. The α4′ helix in loop 9, which can be found in all of the compared amidase structures is not present in AmiP.

**Table S3.** The 30 binding poses calculated by GOLD suite were analyzed according to several criteria: the GoldScore and ChemScore fitness, visually for Zn^2+^ ion coordination, chemical soundness and formed hydrogen bonds. The solutions with teal backgrounds do not show a water molecule in the binding site, whereas the pink marked solutions accommodate a water molecule. The best solution was identified as the most chemical sensible, highest ChemScore fitness and high GoldScore fitness: solution 7 (no water), solution 19 (with water).

**Figure S1.** Multiple sequence alignment of AmiP (PDB 7B3N) with structural homologues using BlastP (Altschul et al., 2020). Representation with ESPript3 (Robert & Gouet, 2014) PDB 4RN7 (Tan et al., n. d.) – N‐acetylmuramoyl‐L‐alanine amidase from *Clostridioides difficile* (UniProtKB Q183J9), PDB 5EMI (Büttner et al., 2016) – AmiC2 from *Nostoc punctiforme* (UniProtKB B2J2S4), PDB 5 J72 (Usenik et al.,, 2017) – Cwp6 from *Clostridioides difficile* (UniProtKB Q183L9), PDB 1JWQ (Yamane et al., 2003) – CwlV from *Paenibacillus polymyxa* subsp. *colistinus* (UniProtKB Q9LCR3), PDB 4M6I (Prigozhin et al., 2013) – Rv3717 from *Mycobacterium tuberculosis* (UniProtKB O69684), PDB 3QAY (Mayer et al., 2011) – CD27L from *Clostridioides difficile* phage ϕCD27 (UniProtKB B6SBV8), PDB 3CZX (Zhang et al., n. d.) – N‐acetylmuramoyl‐L‐alanine amidase from *Neisseria meningitidis* (UniProtKB Q9JZE9). The secondary structural elements of the AmiP structure are shown above the sequences.

**Figure S2.** Positive ion, high resolution LC ESI TOF spectrum of AmiP. The data was generated using a QTof Premier mass spectrometer (Waters) and processed using MassLynx 4.1 and MaxEnt 1 (Waters). The calculated mass of AmiP including the N‐terminal His‐tag is 20,584 Da. With the N‐terminal Met residue cleaved the resulting mass corresponds to 20,453 Da, which is in good agreement with the observed base peak of 20,450 Da.

**Figure S3.** Integrity and purity assessment for AmiP by 4–15% glycine SDS‐PAGE. Electrophoretic profiles are shown of all affinity purification stages, in particular AmiP after elution as well as after dialysis into a reaction buffer. The molecular mass standards are presented in kDa.

**Figure S4.** Thermostability and thermoaggregation of AmiP amidase. (A) Thermostability of AmiP determined by thermal unfolding applying nanoscale differential scanning fluorometry at 20% excitation power with a temperature gradient between 20 and 110°C at a ramp rate of 1°C/min. (B) The first derivative of fluorescence ratio change as a function of temperature. T_m_ – melting temperature. (C) Thermoaggregation of AmiP determined by light scattering applying backreflection technology at 20% excitation power with a temperature gradient between 20–110°C at a ramp rate of 1 °C/min. T_agg_ – mid‐aggregation temperature. Values represent the mean ± standard deviation (n = 3).

**Figure S5.** Scheme of the secondary structure elements (SSE) of Amidase_3 domain folds. In comparison with other Amidase_3 structures (Table S1) the pink SSEs are omitted or significantly shorter in the AmiP structure.

**Figure S6.** Interactions of the highest ranked docking pose of MTP in the AmiP active site. The catalytic Zn^2+^ ion is coordinated by the muramyl carbonyl oxygen and the L‐alanine carbonyl oxygen (yellow punctured lines). Three hydrogen bonds are formed upon muramyl tetrapeptide docking (orange punctured lines).

## Data Availability

Data available on request from the authors.
