# Supplementary material for: AmiP from hyperthermophilic Thermus parvatiensis prophage is a thermoactive and ultrathermostable peptidoglycan lytic amidase
Source: Protein Sci. 2023 Feb 15;32(3):e4585. doi: 10.1002/pro.4585 (PMC9929850; doi:10.1002/pro.4585)
Supplement: Supplementary file 1 — Data S1: Supporting information [file PRO-32-e4585-s001.docx]

**Supplementary Material**

**Figure Captions:**

**Fig**. **S1** Multiple sequence alignment of AmiP (PDB 7B3N) with structural homologues using BlastP (61). Representation with ESPript3 (84) PDB 4RN7 (52) – N-acetylmuramoyl-L-alanine amidase from *Clostridioides* *difficile* (UniProtKB Q183J9), PDB 5EMI (23) – AmiC2 from *Nostoc* *punctiforme* (UniProtKB B2J2S4), PDB 5J72 (11) – Cwp6 from *Clostridioides* *difficile* (UniProtKB Q183L9), PDB 1JWQ (53) – CwlV from *Paenibacillus* *polymyxa* subsp. *colistinus* (UniProtKB Q9LCR3), PDB 4M6I (24) – Rv3717 from *Mycobacterium* *tuberculosis* (UniProtKB O69684), PDB 3QAY (14) – CD27L from *Clostridioides* *difficile* phage ϕCD27 (UniProtKB B6SBV8), PDB 3CZX (54) – N-acetylmuramoyl-L-alanine amidase from *Neisseria* *meningitidis* (UniProtKB Q9JZE9). The secondary structural elements of the AmiP structure are shown above the sequences.

**Fig. S2** Positive ion, high resolution LC ESI TOF spectrum of AmiP. The data was generated using a QTof Premier mass spectrometer (Waters) and processed using MassLynx 4.1 and MaxEnt 1 (Waters). The calculated mass of AmiP including the N-terminal His-tag is 20584 Da. With the N-terminal Met residue cleaved the resulting mass corresponds to 20453 Da, which is in good agreement with the observed base peak of 20450 Da.

**Fig. S3** Integrity and purity assessment for AmiP by 4 – 15 % glycine SDS-PAGE. Electrophoretic profiles are shown of all affinity purification stages, in particular AmiP after elution as well as after dialysis into a reaction buffer. The molecular mass standards are presented in kDa.

**Fig**. **S4** Thermostability and thermoaggregation of AmiP amidase. (A) Thermostability of AmiP determined by thermal unfolding applying nanoscale differential scanning fluorometry at 20 % excitation power with a temperature gradient between 20 – 110 °C at a ramp rate of 1 °C/min. (B) The first derivative of fluorescence ratio change as a function of temperature. T_m_ – melting temperature. (C) Thermoaggregation of AmiP determined by light scattering applying backreflection technology at 20 % excitation power with a temperature gradient between 20 – 110 °C at a ramp rate of 1 °C/min. T_agg_ – mid-aggregation temperature. Values represent the mean ± standard deviation (n = 3).

**Fig**. **S5** Scheme of the secondary structure elements (SSE) of Amidase_3 domain folds. In comparison with other Amidase_3 structures (Table S1) the pink SSEs are omitted or significantly shorter in the AmiP structure.

**Fig**. **S6** Interactions of the highest ranked docking pose of MTP in the AmiP active site. The catalytic Zn^2+^ ion is coordinated by the muramyl carbonyl oxygen and the L-alanine carbonyl oxygen (yellow punctured lines). Three hydrogen bonds are formed upon muramyl tetrapeptide docking (orange punctured lines).
