# Supplementary material for: AmiP from hyperthermophilic Thermus parvatiensis prophage is a thermoactive and ultrathermostable peptidoglycan lytic amidase
Source: Protein Sci. 2023 Feb 15;32(3):e4585. doi: 10.1002/pro.4585 (PMC9929850; doi:10.1002/pro.4585)
Supplement: Supplementary file 2 — Table S1. Amino acid composition of AmiP and homologous Amidases_3 domains as found in Uniprot. [file PRO-32-e4585-s008.docx]

**Table** **S1**. Amino acid composition of AmiP and homologous Amidases_3 domains as found in Uniprot.

| PDB | Uniprot | Name | Residues | Res.  No. | Amino acid number | | | | | | | | | | | | | | | | | | | |
| --- | --- | --- | --- | --- | --- | --- | --- | --- | --- | --- | --- | --- | --- | --- | --- | --- | --- | --- | --- | --- | --- | --- | --- | --- |
|  |  |  |  |  | Ala | Arg | Asn | Asp | Cys | Gln | Glu | Gly | His | Ile | Leu | Lys | Met | Phe | Pro | Ser | Thr | Trp | Tyr | Val |
| 7B3N | H7GE39 | AmiP | 11 - 147 | 137 | 17 | 12 | 1 | 8 | 0 | 2 | 7 | 15 | 4 | 5 | 13 | 1 | 2 | 5 | 11 | 9 | 7 | 1 | 6 | 11 |
| 4RN7 (55) | Q183J9 | amidase | 180 - 292 | 113 | 9 | 4 | 10 | 4 | 1 | 6 | 11 | 7 | 1 | 10 | 9 | 6 | 1 | 2 | 2 | 8 | 8 | 1 | 3 | 10 |
| 5EMI (28) | B2J2S4 | AmiC2 | 502 - 611 | 110 | 11 | 11 | 9 | 5 | 0 | 3 | 8 | 6 | 1 | 8 | 7 | 3 | 3 | 2 | 3 | 7 | 6 | 0 | 7 | 10 |
| 5J72 (16) | Q183L9 | Cwp6 | 552 - 675 | 124 | 7 | 4 | 14 | 8 | 1 | 1 | 5 | 10 | 1 | 11 | 9 | 15 | 3 | 3 | 2 | 6 | 12 | 0 | 6 | 6 |
| 1JWQ (56) | Q9LCR3 | CwlV | 385 - 493 | 109 | 14 | 5 | 8 | 4 | 0 | 3 | 7 | 8 | 3 | 5 | 6 | 5 | 2 | 6 | 2 | 9 | 8 | 0 | 5 | 9 |
| 4M6I (29) | O69684 | Rv3717 | 110 - 231 | 122 | 20 | 7 | 10 | 5 | 0 | 7 | 3 | 13 | 2 | 6 | 12 | 3 | 4 | 2 | 8 | 9 | 0 | 0 | 5 | 6 |
| 3QAY (19) | B6SBV8 | CD27L | 70 - 174 | 105 | 6 | 4 | 7 | 6 | 2 | 1 | 7 | 11 | 2 | 6 | 14 | 12 | 0 | 3 | 2 | 7 | 3 | 0 | 6 | 6 |
| 3CZX (57) | Q9JZE9 | amidase | 5 - 173 | 169 | 23 | 9 | 10 | 13 | 3 | 4 | 5 | 17 | 3 | 11 | 12 | 13 | 2 | 6 | 5 | 7 | 13 | 3 | 2 | 8 |

Thermolabile

Thermostable
