# Supplementary material for: AmiP from hyperthermophilic Thermus parvatiensis prophage is a thermoactive and ultrathermostable peptidoglycan lytic amidase
Source: Protein Sci. 2023 Feb 15;32(3):e4585. doi: 10.1002/pro.4585 (PMC9929850; doi:10.1002/pro.4585)
Supplement: Supplementary file 3 — Table S2. Comparison of the secondary structure elements of AmiP with homologous lytic amidases by least squares superposition and subsequent visual assessment. The α4′ helix in loop 9, which can be found in all of the compared amidase structures is not present in AmiP. [file PRO-32-e4585-s009.docx]

**Table** **S2**. Comparison of the secondary structure elements of AmiP with homologous lytic amidases by least squares superposition and subsequent visual assessment. The α4' helix in loop 9, which can be found in all of the compared amidase structures is not present in AmiP.

| PDB | Name | Residues | Res.  No. | Secondary structure elements (SSE) | | | | | | | | | | | | | | | | | | | |
| --- | --- | --- | --- | --- | --- | --- | --- | --- | --- | --- | --- | --- | --- | --- | --- | --- | --- | --- | --- | --- | --- | --- | --- |
|  |  |  |  | Helix | | | | | Strand | | | | | | Loop | | | | | | | | |
|  |  |  |  | α1 | α2 | α3 | α4' | α4 | β1 | β2 | β3 | β4 | β5 | β6 | l1 | l2 | l3 | l4 | l5 | l6 | l7 | l8 | l9 |
| 4RN7 (Tan et al., n.d.) | amidase | 114 – 299 | 186 | 0 | 0 | + | P | 0 | + | + | + | + | + | + | 0 | 0 | 0 | 0 | + | + | 0 | - | + |
| 5EMI (Büttner et al., 2016) | AmiC2 | 435 – 614 | 180 | 0 | 0 | + | P | 0 | + | + | 0 | + | + | 0 | 0 | 0 | 0 | 0 | + | - | 0 | - | + |
| 5J72 (Usenik et al., 2017 | Cwp6 | 457 – 647 | 191 | 0 | 0 | + | P | 0 | + | + | + | 0 | + | + | + | 0 | 0 | 0 | + | + | 0 | 0 | + |
| 1JWQ (Yamane et al., 2003) | CwlV | 1 – 179 | 179 | 0 | 0 | + | P | 0 | + | + | + | + | + | + | 0 | + | 0 | 0 | + | 0 | 0 | - | + |
| 4M6I (Prigozhin et al., 2013) | Rv3717 | 24 – 237 | 214 | 0 | 0 | + | P | 0 | + | + | + | + | + | + | 0 | 0 | 0 | 0 | 0 | 0 | + | - | + |
| 3QAY (Mayer et al., 2011) | CD27L | 1 – 180 | 180 | 0 | + | + | P | - | + | + | + | + | + | + | + | 0 | 0 | 0 | + | - | 0 | - | + |
| 3CZX (Zhang et al., n.d.) | amidase | 1 – 182 | 182 | 0 | 0 | + | P | 0 | 0 | 0 | - | 0 | 0 | - | 0 | 0 | 0 | 0 | + | - | + | - | + |

0 – SSE same length as in AmiP; + – SSE longer compared to AmiP; - – SSE shorter compared to AmiP.

P – SSE present.
