# Supplementary material for: AmiP from hyperthermophilic Thermus parvatiensis prophage is a thermoactive and ultrathermostable peptidoglycan lytic amidase
Source: Protein Sci. 2023 Feb 15;32(3):e4585. doi: 10.1002/pro.4585 (PMC9929850; doi:10.1002/pro.4585)
Supplement: Supplementary file 4 — Table S3. The 30 binding poses calculated by GOLD suite were analyzed according to several criteria: the GoldScore and ChemScore fitness, visually for Zn2+ ion coordination, chemical soundness and formed hydrogen bonds. The solutions with teal backgrounds do not show a water molecule in the binding site, whereas the pink marked solutions accommodate a water molecule. The best solution was identified as the most chemical sensible, highest ChemScore fitness and high GoldScore fitness: solution 7 (no water), solution 19 (with water). [file PRO-32-e4585-s010.docx]

**Table S3.** The 30 binding poses calculated by GOLD suite were analysed according to several criteria: the GoldScore and ChemScore fitness, visually for Zn^2+^ ion coordination, chemical soundness and formed hydrogen bonds. The solutions with teal backgrounds do not show a water molecule in the binding site, whereas the pink marked solutions accommodate a water molecule. The best solution was identified as the most chemical sensible, highest ChemScore fitness and high GoldScore fitness: solution 7 (no water), solution 19 (with water).

| Solution | GoldScore | ChemScore | Water | Zn^2+^ ion coordination | Hydrogen bonds | Rejection reason |
| --- | --- | --- | --- | --- | --- | --- |
| 1 | 17.3 | -29.78 | N | D-Gln CO – Zn | 4, 1 short | scores |
| 2 | 39.3 | -23.9 | N | L-Ala CO – Zn | 5 | scores |
| 3 | 48.5 | -22.9 | Y | sugar – **water** - Zn | 3 |  |
| 4 | 0.08 | -37.1 | N | sugar – Zn | 5 | scores |
| 5 | 47.2 | -20.2 | Y | L-Ala CO - **water** - Zn | 4 |  |
| 6 | 65.2 | -25.0 | N | L-Ala CO – Zn | 4 |  |
| 7 | 56.4 | -13.2 | N | Mur CO – Zn | 3 |  |
| 8 | 33.4 | -20.3 | Y | Mur CO – **water** – Zn | 3 | scores |
| 9 | 35.2 | -28.8 | N | L-Ala CO – Zn | 4, 1 too short | scores |
| 10 | 23.6 | -39.1 | N | sugar – Zn | 7 | scores |
| 11 | 3.8 | -20.6 | Y | sugar - **water** – Zn | 5 | scores |
| 12 | 44.0 | -22.5 | N | far from Zn | - | clashes |
| 13 | -10.6 | -23.4 | N | Glu C - Zn | 2 | Zn^2+^ coordination |
| 14 | 55.9 | -15.6 | N | Glu SC - Zn | 2 |  |
| 15 | 13.2 | -45.5 | Y | Glu N - **water** - Zn | 6 | scores |
| 16 | 28.9 | -38.3 | Y | Glu CO - **water** - Zn | 3 | scores |
| 17 | 48.5 | -21.5 | N | Ala CO - Zn | 2 |  |
| 18 | -2.8 | -32.4 | Y | D-Ala - **water** – Zn | - | clashes |
| 19 | 42.6 | -15.7 | Y | Ala CO - **water** - Zn | 6 |  |
| 20 | 23.2 | -27.0 | N | Ala CO - Zn | 4 | scores |
| 21 | 14.4 | -24.0 | Y | Mur CO - **water** - Zn | 6 | scores |
| 22 | 7.9 | -39.6 | N | clashes | - | clashes |
| 23 | 36.6 | -14.5 | N | Mur CO - Zn | 0 | no hydrogen bonds |
| 24 | 45.2 | -19.6 | Y | **water** – Zn | 8 |  |
| 25 | 42.7 | -24.3 | N | Ala CO - Zn | 3 |  |
| 26 | -21.8 | -37.6 | Y | sugar - **water** - Zn | 4 | Zn^2+^ coordination |
| 27 | 48.4 | -26.6 | Y | Mur CO - **water** - Zn | 6 |  |
| 28 | 59.4 | -16.4 | N | Ala CO - Zn | 4 |  |
| 29 | 40.9 | -14.3 | Y | Orn CO - **water**- Zn | 6 |  |
| 30 | -1.2 | -30.3 | N | C- Zn | 3 | Zn^2+^ coordination |
