# Supplementary figures and images for: AmiP from hyperthermophilic Thermus parvatiensis prophage is a thermoactive and ultrathermostable peptidoglycan lytic amidase
Source: Protein Sci. 2023 Feb 15;32(3):e4585. doi: 10.1002/pro.4585 (PMC9929850; doi:10.1002/pro.4585)

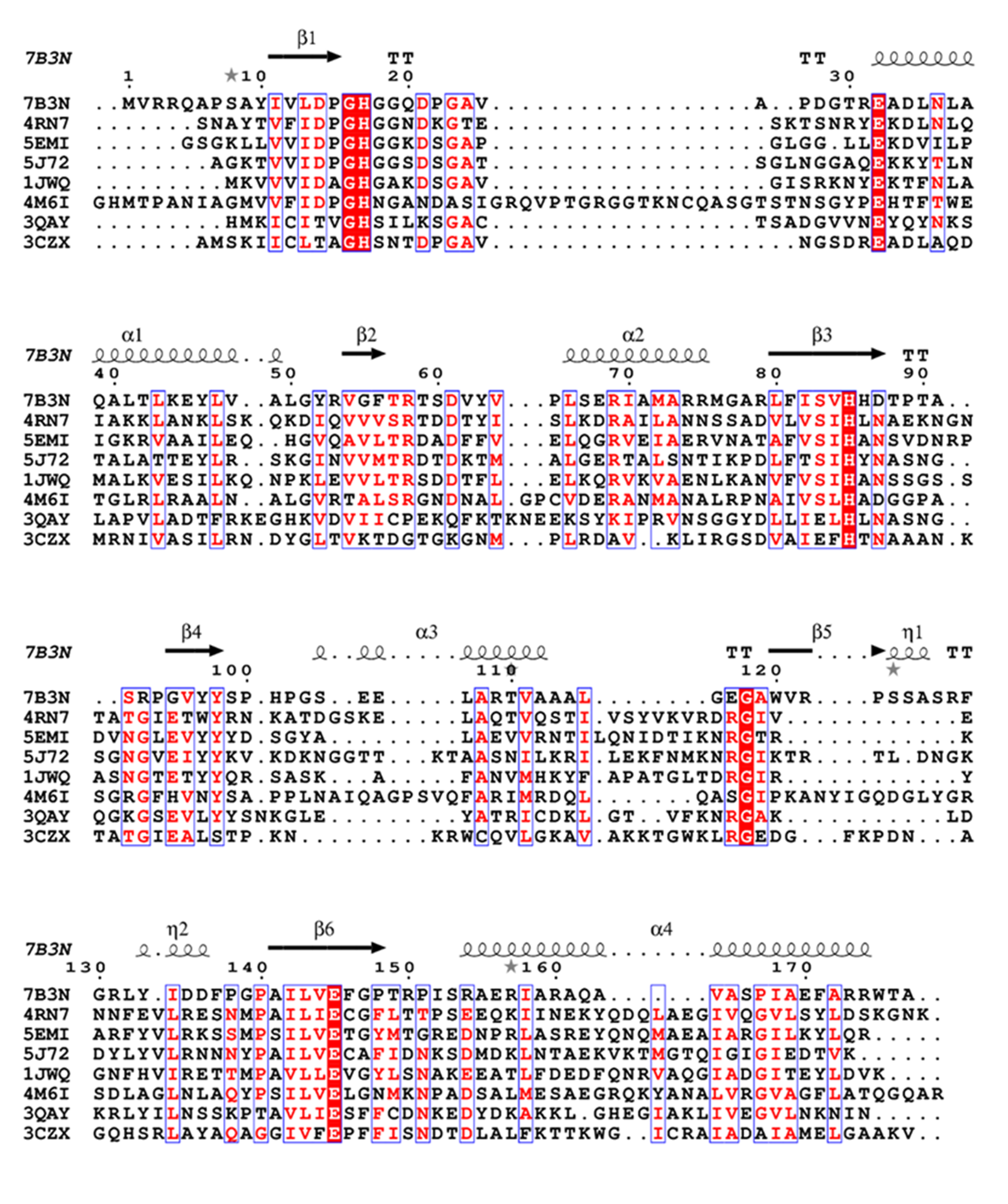

Supplement: Supplementary file 5 — Figure S1. Multiple sequence alignment of AmiP (PDB 7B3N) with structural homologues using BlastP (Altschul et al., 2020). Representation with ESPript3 (Robert & Gouet, 2014) PDB 4RN7 (Tan et al., n. d.) – N‐acetylmuramoyl‐L‐alanine amidase from Clostridioides difficile (UniProtKB Q183J9), PDB 5EMI (Büttner et al., 2016) – AmiC2 from Nostoc punctiforme (UniProtKB B2J2S4), PDB 5 J72 (Usenik et al.,, 2017) – Cwp6 from Clostridioides difficile (UniProtKB Q183L9), PDB 1JWQ (Yamane et al., 2003) – CwlV from Paenibacillus polymyxa subsp. colistinus (UniProtKB Q9LCR3), PDB 4M6I (Prigozhin et al., 2013) – Rv3717 from Mycobacterium tuberculosis (UniProtKB O69684), PDB 3QAY (Mayer et al., 2011) – CD27L from Clostridioides difficile phage ϕCD27 (UniProtKB B6SBV8), PDB 3CZX (Zhang et al., n. d.) – N‐acetylmuramoyl‐L‐alanine amidase from Neisseria meningitidis (UniProtKB Q9JZE9). The secondary structural elements of the AmiP structure are shown above the sequences. [file PRO-32-e4585-s007.tif]

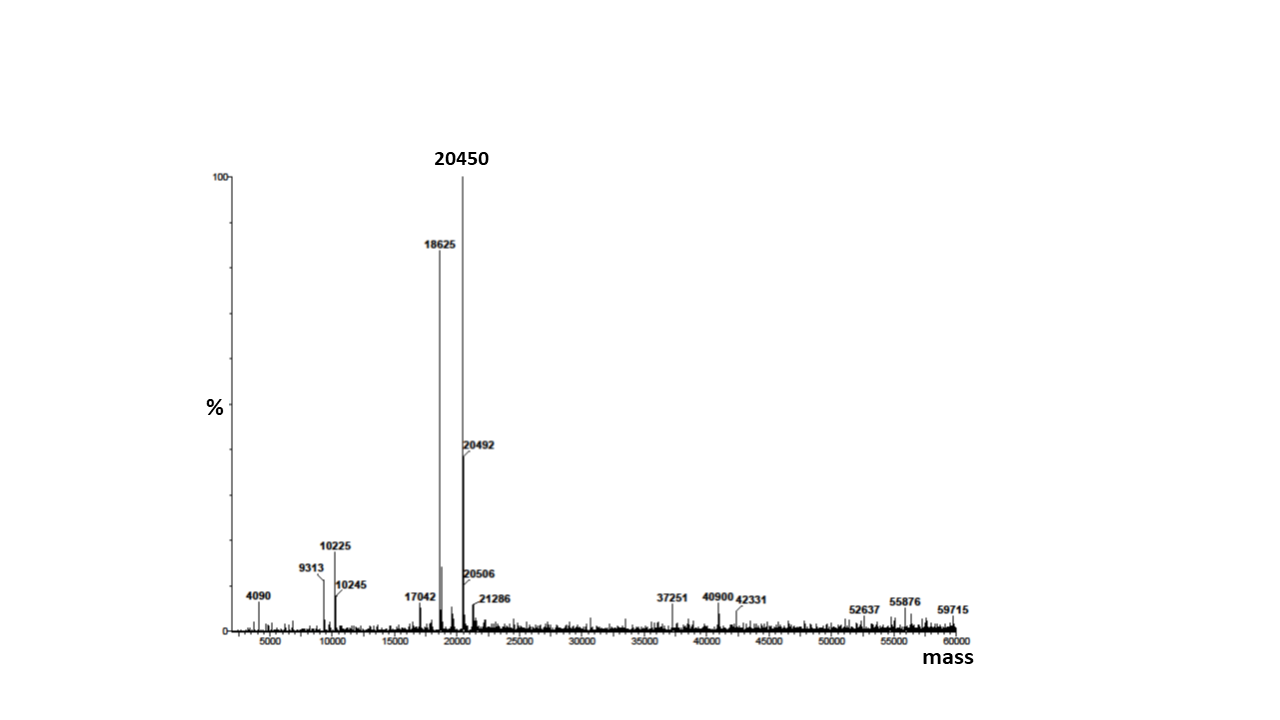

Supplement: Supplementary file 6 — Figure S2. Positive ion, high resolution LC ESI TOF spectrum of AmiP. The data was generated using a QTof Premier mass spectrometer (Waters) and processed using MassLynx 4.1 and MaxEnt 1 (Waters). The calculated mass of AmiP including the N‐terminal His‐tag is 20,584 Da. With the N‐terminal Met residue cleaved the resulting mass corresponds to 20,453 Da, which is in good agreement with the observed base peak of 20,450 Da. [file PRO-32-e4585-s004.tif]

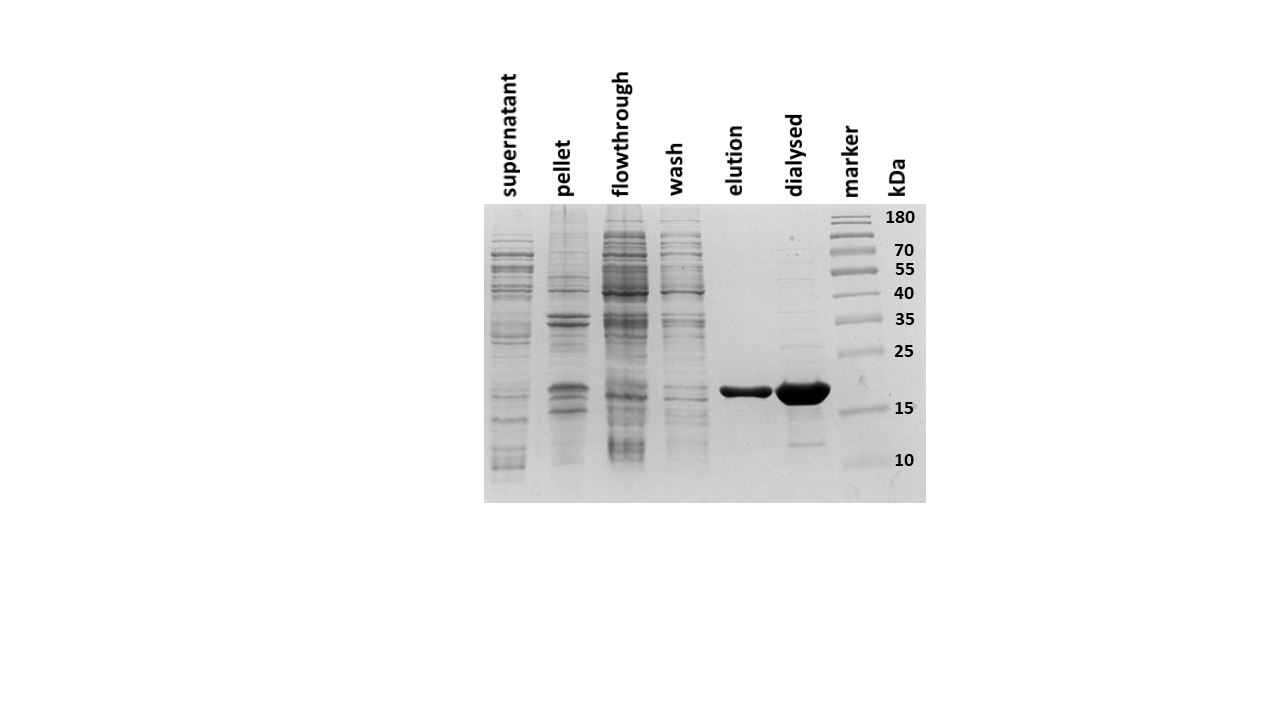

Supplement: Supplementary file 7 — Figure S3. Integrity and purity assessment for AmiP by 4–15% glycine SDS‐PAGE. Electrophoretic profiles are shown of all affinity purification stages, in particular AmiP after elution as well as after dialysis into a reaction buffer. The molecular mass standards are presented in kDa. [file PRO-32-e4585-s003.tif]

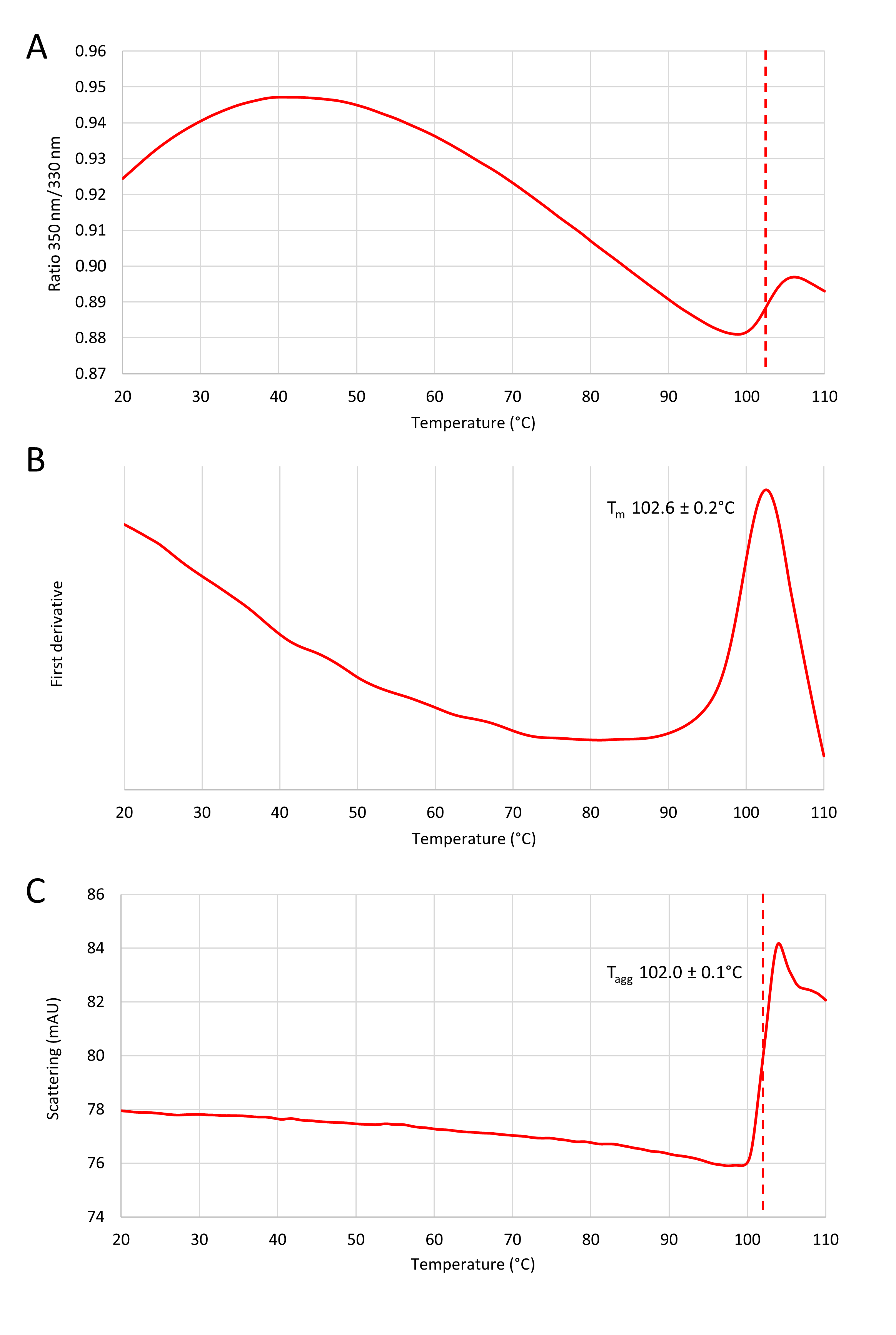

Supplement: Supplementary file 8 — Figure S4. Thermostability and thermoaggregation of AmiP amidase. (A) Thermostability of AmiP determined by thermal unfolding applying nanoscale differential scanning fluorometry at 20% excitation power with a temperature gradient between 20 and 110°C at a ramp rate of 1°C/min. (B) The first derivative of fluorescence ratio change as a function of temperature. Tm – melting temperature. (C) Thermoaggregation of AmiP determined by light scattering applying backreflection technology at 20% excitation power with a temperature gradient between 20–110°C at a ramp rate of 1 °C/min. Tagg – mid‐aggregation temperature. Values represent the mean ± standard deviation (n = 3). [file PRO-32-e4585-s005.tiff]

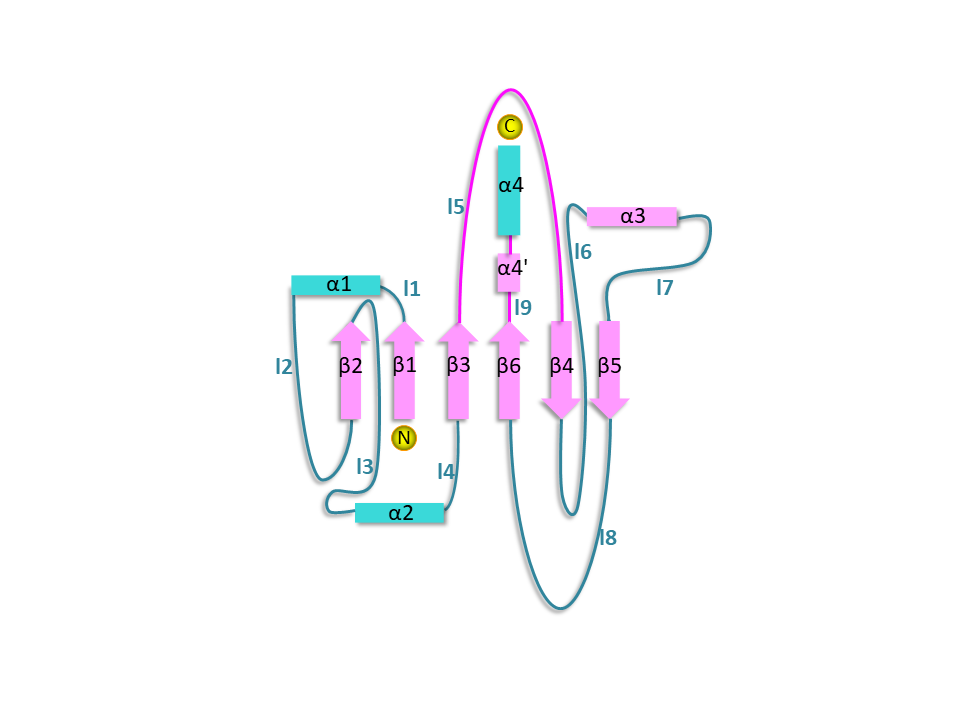

Supplement: Supplementary file 9 — Figure S5. Scheme of the secondary structure elements (SSE) of Amidase_3 domain folds. In comparison with other Amidase_3 structures (Table S1) the pink SSEs are omitted or significantly shorter in the AmiP structure. [file PRO-32-e4585-s006.tif]

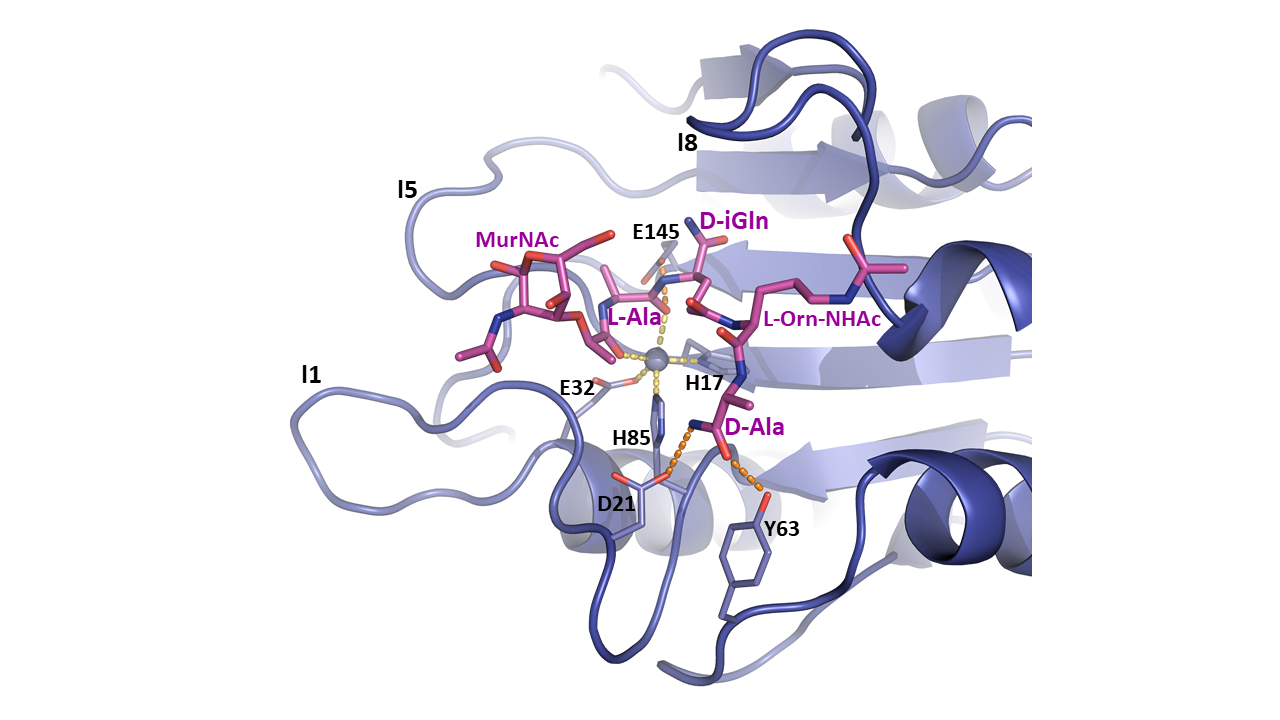

Supplement: Supplementary file 10 — Figure S6. Interactions of the highest ranked docking pose of MTP in the AmiP active site. The catalytic Zn2+ ion is coordinated by the muramyl carbonyl oxygen and the L‐alanine carbonyl oxygen (yellow punctured lines). Three hydrogen bonds are formed upon muramyl tetrapeptide docking (orange punctured lines). [file PRO-32-e4585-s002.tif]
